# K63-Linked Ubiquitination Targets *Toxoplasma gondii* for Endo-lysosomal Destruction in IFNγ-Stimulated Human Cells

**DOI:** 10.1371/journal.ppat.1006027

**Published:** 2016-11-22

**Authors:** Barbara Clough, Joseph D. Wright, Pedro M. Pereira, Elizabeth M. Hirst, Ashleigh C. Johnston, Ricardo Henriques, Eva-Maria Frickel

**Affiliations:** 1 Host-Toxoplasma Interaction Laboratory, The Francis Crick Institute, United Kingdom; 2 MRC Laboratory for Molecular Cell Biology and Department of Cell and Developmental Biology, University College London, United Kingdom; University of New Mexico, UNITED STATES

## Abstract

*Toxoplasma gondii* is the most common protozoan parasitic infection in man. Gamma interferon (IFNγ) activates haematopoietic and non-haematopoietic cells to kill the parasite and mediate host resistance. IFNγ-driven host resistance pathways and parasitic virulence factors are well described in mice, but a detailed understanding of pathways that kill *Toxoplasma* in human cells is lacking. Here we show, that contrary to the widely held belief that the *Toxoplasma* vacuole is non-fusogenic, in an immune-stimulated environment, the vacuole of type II *Toxoplasma* in human cells is able to fuse with the host endo-lysosomal machinery leading to parasite death by acidification. Similar to murine cells, we find that type II, but not type I *Toxoplasma* vacuoles are targeted by K63-linked ubiquitin in an IFNγ-dependent manner in non-haematopoetic primary-like human endothelial cells. Host defence proteins p62 and NDP52 are subsequently recruited to the type II vacuole in distinct, overlapping microdomains with a loss of IFNγ-dependent restriction in p62 knocked down cells. Autophagy proteins Atg16L1, GABARAP and LC3B are recruited to <10% of parasite vacuoles and show no parasite strain preference, which is consistent with inhibition and enhancement of autophagy showing no effect on parasite replication. We demonstrate that this differs from HeLa human epithelial cells, where type II *Toxoplasma* are restricted by non-canonical autophagy leading to growth stunting that is independent of lysosomal acidification. In contrast to mouse cells, human vacuoles do not break. In HUVEC, the ubiquitinated vacuoles are targeted for destruction in acidified LAMP1-positive endo-lysosomal compartments. Consequently, parasite death can be prevented by inhibiting host ubiquitination and endosomal acidification. Thus, K63-linked ubiquitin recognition leading to vacuolar endo-lysosomal fusion and acidification is an important, novel virulence-driven *Toxoplasma* human host defence pathway.

## Author Summary

*Toxoplasma gondii* is an intracellular parasite that can invade nucleated cells of any warm-blooded animal into a compartment known as a parasitophorous vacuole (PV). The production of gamma interferon (IFNγ) drives the restriction and killing of *Toxoplasma*. It is not fully known how the parasite inside the PV is eliminated in human cells, although its fate depends on the cell type into which it invades. In IFNγ-stimulated epithelial HeLa cells for instance growth of type II parasites is restricted 24h post-infection by employing the cellular autophagy pathway. Distinctly, we show here that in human endothelial cells the parasite is destroyed by fusion of the PV with the cell’s endo-lysosomal pathway as early as 6h post-infection. This process, which is at odds with the normally non-fusogenic nature of the PV, is dependent on IFNγ. Parasite death follows Lysine63-linked ubiquitination of the PV and is specific to type II *Toxoplasma*. Our results demonstrate for the first time that vacuolar acidification leading to parasite death is central to controlling infection by *Toxoplasma* in human endothelial cells.

## Introduction

Host cells invaded by intracellular pathogens have to mount a rapid recognition and cell-autonomous defence program to curb the replication of the intruder [1]. The cytokine gamma interferon (IFNγ) can stimulate cell-autonomous defence in immune or non-immune cells and is produced early during infection with many intracellular pathogens, including the protozoan parasite *Toxoplasma gondii* [2].

Worldwide, human *Toxoplasma* infections are estimated at 30% and the parasite can infect all warm-blooded animals. Human infections are mostly asymptomatic, but the parasite establishes a lifelong chronic infection in the form of cysts in brain and muscle tissue. Ocular disease is a complication for both the immunocompetent and immunocompromised, while serious illness and death are possibilities in the immunocompromised and the developing foetus of pregnant women. *Toxoplasma* strains in North America and Europe are mostly of the types I, II and III, with type I strains classified as highly virulent with an LD100 of 1 parasite in mice, and type II and III strains being less virulent in mice, with an LD50 greater than 1000 parasites/mouse [3,4].

*Toxoplasma gondii* actively invades any nucleated host cell where it resides and replicates within a nonfusogenic parasitophorous vacuole (PV) [5–7] and without immune pressure resists acidification [5,8]. Many defence mechanisms against *Toxoplasma* have been identified in macrophages of mice and man. For both organisms, CD40 ligation stimulates autophagy and fusion of the PV with lysosomes [9] and activation of the purinergic receptor P2X7R leads to killing of the parasite [10]. Interferon-induced production of nitric oxide plays a role in chronic infection in mice [11], but not in human macrophages [12]. In mice, Atg5 has previously been shown to be important in the murine host immune response to *Toxoplasma* [13,14].

IFNγ not only stimulates macrophages but non-immune cells, and in chimeric mice, gamma interferon receptor is critical in both haematopoetic and nonhaematopoetic cell types [15]. In mice, the most important interferon-inducible effector mechanisms are the IRGs and p65 guanylate-binding proteins (GBPs), both of which localise to the PV and disrupt the membrane of the vacuole. The autophagy proteins Atg7, Atg3, and the Atg12-Atg5-Atg16L1 complex were reported to target IRGs and GBPs to the PV of *Toxoplasma* for disruption in mouse cells [16]. Similarly, Atg7 and Atg16L1 deficient MEFs were impaired in the recruitment of IRGs and GBPs to the PV [17]. Unlike mice, humans lack IFNγ-inducible versions of IRGs and do not recruit GBP1 to the vacuole [18]. However, we have reported that GBP1 mediates an early restriction of *Toxoplasma* that is not dependent upon vacuolar localisation [18].

Of interest, it has long been appreciated that in human IFNγ-stimulated fibroblasts, both type I as well as type II parasite replication is controlled [19–21]. This has been attributed to nutrient starvation driven by the IFNγ-inducible indoleamine 2,3-dioxygenase (IDO1) which degrades tryptophan for which *Toxoplasma* is auxotrophic [20]. While we have previously found that restriction of *Toxoplasma* is solely dependent on IDO1 in HeLa cells, in fibroblasts, interferon-dependent restriction is only partially mediated by IDO1 [22]. In fibroblasts, it is not overtly dependent on autophagy as assessed by Atg5 knockdown [22], nor is the common *Toxoplasma* mouse virulence factor Rop18 involved [21]. Additionally, IDO1-mediated restriction occurs in fibroblasts but not in endothelial or epithelial cells [23,24]. Human endothelial cells have been shown to restrict *Toxoplasma* in an autophagy- and lysosome-independent manner [25] and human fibroblast-like cells (HAP1) equally do not restrict *Toxoplasma* by autophagy [17]. A recent report has attributed the restriction of type II and III *Toxoplasma* in HeLa cells to a non-canonical acidification-independent autophagy pathway, requiring p62 and NDP52 [26]. It is thus apparent that multiple mechanisms of interferon-induced cell-autonomous resistance must exist in different human non-haematopoetic cells, but the nature of these is unclear.

Other intracellular pathogens such as the bacteria *Salmonella typhimurium* are cleared by autophagy after an initial cellular recognition event that marks the bacteria or the bacterial vacuole with host cellular ubiquitin. Ubiquitination recruits autophagy adaptor proteins NDP52, p62, and optineurin, which in turn bind to LC3 that recruits the autophagic double membrane [27,28] leading to bacterial killing by acidification [29]. For control of *Shigella flexneri* infection, however, p62 and NDP52 recruitment has been shown to be inter-dependent and reliant on septin caging and actin polymerization. Other cytosolic bacteria such as the *Listeria monocytogenes* ActA mutant, that are susceptible to ubiquitination, p62 recruitment and septin caging, do not display the same dependence on septin and actin, suggesting p62 and NDP52 can direct different pathways of selective autophagy [30]. The hallmark event in all these pathways is the ubiquitination, with different ubiquitin linkages dictating different cellular responses [31].

Here we report the cell-autonomous killing of type II *Toxoplasma gondii* in human non-haematopoetic cells by fusion with the host’s endo-lysosomal system. We show that, in contrast to mouse cells, the PV in IFNγ-stimulated human cells does not rupture, but rather becomes LAMP1- and LysoTracker-positive indicating fusion with the endo-lysosomal system. Consequently, the parasites are acidified and die within the PV. IFNγ-driven K63-linked ubiquitination of the PV is a prerequisite of parasite death with the subsequent recruitment of NDP52 and p62 in overlapping microdomains. We do not find an overt dependence on autophagy, as down-regulation of the autophagy pathway by Atg16L1 knock-down does not rescue parasite growth. However, consistent with their role in parasite killing, inhibition of host ubiquitination, and vacuolar/endosomal acidification, enhance parasite viability under IFNγ-stimulated conditions.

## Results

### IFNγ drives K63-linked ubiquitination of the type II *Toxoplasma* vacuole

**Fig 1:**
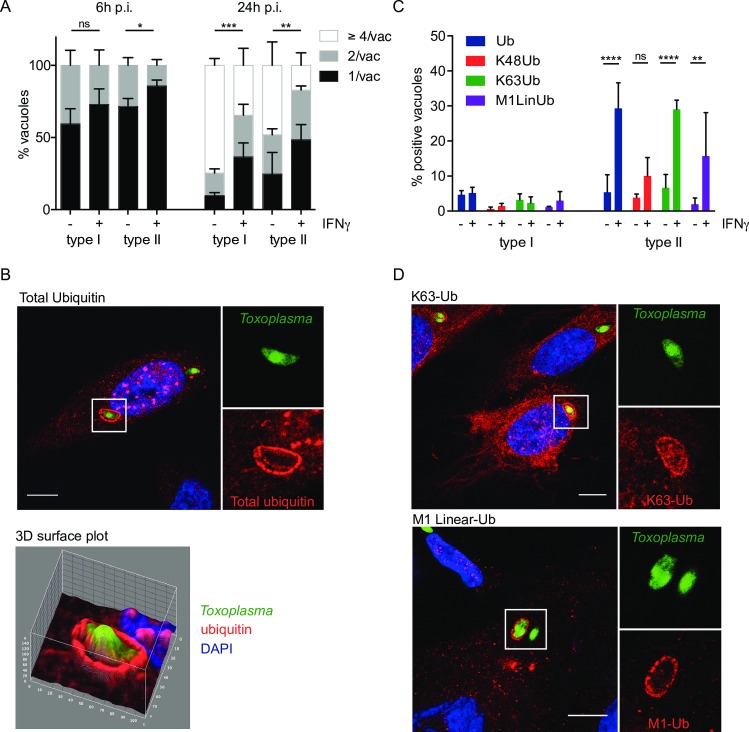
IFNγ-mediated K63-linked ubiquitination of type II *Toxoplasma* vacuoles. (A) Replication of both type I and type II *Toxoplasma* is diminished in IFNγ-stimulated HUVEC at 6h and more substantially at 24h post invasion. HUVEC were infected (-/+ stimulation with 50 units/ml IFNγ for 18h) with type I or type II parasites for 6h and 24h. Results from ≥3 experiments are shown. Significance was calculated using 2way ANOVA, *, p≤0.05, **, p≤0.01, ***, p≤0.001 and ns, not significant. (B) Ubiquitin is recruited to the PV of type II *Toxoplasma* in HUVEC. HUVEC were stimulated with 50 units/ml IFNγ for 18h before infecting with type II *Toxoplasma* for 2.5h. Confocal image of a representative vacuole is shown. Scale bar 10μm. The ubiquitin staining is mainly at the PV and not substantially on the parasite. 3D surface profile (Image J) of the pixel intensity over a ubiquitin-stained vacuole containing type II *Toxoplasma*, on a confocal section. Ubiquitin intensity (red) and parasite (green) are shown. (C) Ubiquitin around the type II *Toxoplasma* vacuole is mostly of the K63 linkage. Antibodies specific for ubiquitin linkages K63, K48 and M1 linear were used alongside an antibody staining total ubiquitin. Quantitation of the indicated linkage-specific ubiquitin-positive PVs, at 2.5h p.i., is shown. The mean of 3 experiments is shown. Significance was calculated using 2way ANOVA, **, p≤0.01, ****, p≤ 0.0001 and ns, not significant. (D) K63-linked and M1-linear ubiquitin accumulated at the type II *Toxoplasma* PV. Confocal images were taken of HUVEC stimulated with 50 units/ml IFNγ for 18h, before infecting with type II *Toxoplasma* for 2.5h. Infected cells were then fixed and stained with linkage-specific ubiquitin antibodies. Representative images are shown. Scale bar 10μm.

Human endothelial cells are known to restrict *Toxoplasma* type I growth upon stimulation with gamma interferon [23]. In order to explore the kinetics and strain-dependence of IFNγ-mediated restriction in HUVEC, we enumerated how many parasites were contained per vacuole at 6h and 24h post-infection for both type I and type II parasites. We found that both parasite strains were already restricted in their replication at 6h with increasing significance at 24h (Fig 1A).

Ubiquitin recognition of intracellular bacteria has been shown to be the trigger for critical host effector mechanisms mediating cell-autonomous pathogen restriction [32]. We therefore asked if cellular ubiquitin is recruited to the vicinity of *Toxoplasma* in the presence and absence of IFNγ. Total ubiquitin was assessed by immunofluorescence using the ubiquitin antibody, FK2, which recognizes K29, K48 and K63-linked mono- and poly-ubiquitin chains. A representative confocal microscopy picture of total ubiquitin recruited to the PV of type II *Toxoplasma* is shown in Fig 1B. In HUVEC, stimulated with IFNγ and infected with type II *Toxoplasma*, 30% of PVs were positive for ubiquitin (Fig 1C). Less than 5% PV ubiquitin recruitment was seen upon infection with type I *Toxoplasma* or in the absence of IFNγ. To determine whether the ubiquitin was localised to the vacuole or the parasite, a 3D surface intensity plot was constructed on a confocal plane of the intracellular parasite, and z-stack projections recorded through all confocal planes (Fig 1B and S1 and S2 Figs). A clear separation of fluorescence intensity was observed between the vacuole (red) and parasite (green) in cells stained for total ubiquitin (Fig 1B). Total ubiquitin staining appeared homogenous throughout the surface of PV containing type II parasites (Fig 1B and S1 and S2 Figs).

In murine cells, the virulence factors Rop16 and Rop18 have been shown to modulate STAT signalling, cytokine production and IRG and GBP recruitment [33–40]. In contrast, in HFFs, ROP18 does not impact the host’s cell-autonomous killing ability with parasitic ROP5 expression levels being a minimal defence determinant [21]. We thus asked if the murine virulence factors ROP16 and ROP18 could prevent ubiquitin recognition of the type II PV in HUVEC. Expressing virulence factors ROP16 or ROP18 in type II or III parasites had no impact on the levels of ubiquitin decoration of the PVs (S3 Fig).

As the type of ubiquitin linkage determines the different fates of target proteins [31], we next examined if the ubiquitin recruited to the PV was of a particular linkage. Antibodies directed against M1 linear, K48 and K63-linked ubiquitin were tested. Both K63-linked and M1-linear ubiquitin stained the PV of type II *Toxoplasma*, similarly coating the full circumference of the vacuole (Fig 1D). Quantitation of the PVs coated with M1 linear, K48 and K63-linked ubiquitin clearly indicated that the majority of ubiquitin localising to the PV was in the form of K63-linked poly-ubiquitin chains with 25–30% of type II PVs staining positive (Fig 1C). A significant IFNγ-dependent increase in PVs staining for M1 linear ubiquitin was observed (Fig 1C), with K48-linked ubiquitin recruitment not significantly different from background levels of total ubiquitin staining. These results demonstrate that during infection with type II, as opposed to type I *Toxoplasma*, mainly K63-linked ubiquitin chains are recruited to the PV in an IFNγ-dependent fashion

### Ubiquitin-binding proteins p62 and NDP52 form microdomains on type II *Toxoplasma* PVs

**Fig 2:**
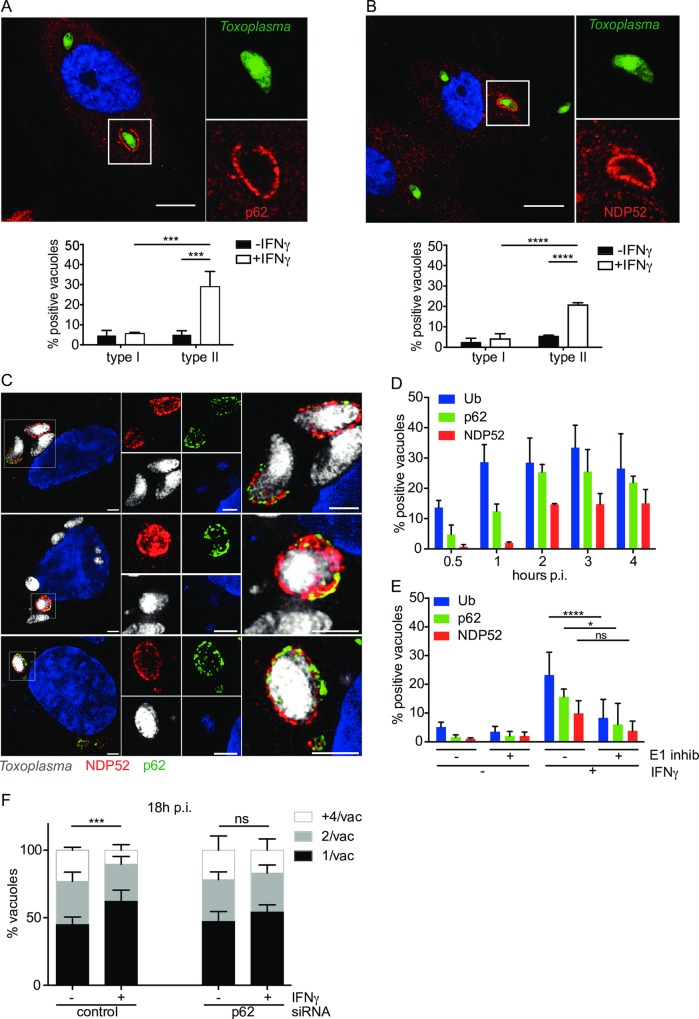
p62 and NDP52 are recruited to microdomains on the type II *Toxoplasma* PV in dependence of ubiquitination. (A) p62 accumulates specifically at the type II *Toxoplasma* PV in IFNγ-stimulated HUVEC. Confocal images were taken of IFNγ-stimulated HUVEC infected with type II *Toxoplasma* for 2.5h, fixed and stained with p62 antibody and a representative image is shown. Scale bar 10μm. Quantitation of p62-positive PVs under the indicated conditions is shown, 2.5h p.i. The mean of 3 experiments is shown. Significance was calculated using 2way ANOVA, ***, p ≤ 0.001. (B) NDP52 accumulates specifically at the type II *Toxoplasma* PV in IFNγ-stimulated HUVEC. Confocal images were taken of HUVEC infected with type II *Toxoplasma* for 2.5h, fixed and stained with NDP52 antibody and a representative image is shown. Scale bar 10μm. Quantitation of NDP52-positive PVs under the indicated conditions is shown, 2.5h p.i.. The mean of 3 experiments is shown. Significance was calculated using 2way ANOVA, ****, p≤ 0.0001. (C) p62 and NDP52 occupy overlapping microdomains at the type II *Toxoplasma* PV in IFNγ-stimulated HUVEC. Superresolution Structured Illumination Microscopy images of 3 representative vacuoles demonstrated mostly a patchy and distinct localisation of p62 and NDP52 with some small overlapping microdomains. Scale bar 2μm. (D) Host defence proteins p62 and NDP52 are recruited to the PV subsequent to ubiquitin binding to the PV. Ubiquitin accumulates on the type II PV within 30min-1h p.i., reaching a maximum already at 1h. p62 sequentially follows the ubiquitin recruitment, with a maximum at 2–4h p.i. and NDP52, appearing last to maximum levels at 2h p.i and is maintained until at least 4h p.i. Quantitation of recruitment-positive type II PVs, at the indicated time-points p.i. is shown. The mean of ≥3 experiments is shown. (E) p62 and NDP52 recruitment to the PV depends upon the ubiquitination of the PV. Inhibition of ubiquitination in HUVEC with the E1 inhibitor UBEI-41 leads to a reduction in ubiquitin as well as p62 and NDP52 at the vacuole of type II *Toxoplasma*. IFNγ-stimulated HUVEC were pre-incubated with 50μM UBEI-41 for 2h before washing and infecting with type II *Toxoplasma* for 2.5h. Cells were stained for ubiquitin, p62, NDP52 and positive staining recorded. The mean of ≥3 experiments is shown. Significance was calculated using 2way ANOVA, *, p ≤ 0.05, ****, p ≤ 0.0001 and ns, not significant. (F) Knockdown of p62 by siRNA leads to the loss of IFNγ-restriction of type II *Toxoplasma*. siRNA nucleofection of p62 was used to knock down the protein in HUVEC, comparing to a control siRNA. 24h after siRNA nucleofection, the cells were stimulated with 50units/ml IFNγ for a further 24h. Infection with type II *Toxoplasma* was then allowed to proceed for 18h and the effect on replication recorded in fixed cells by scoring the numbers of parasites/vacuole in >100 vacuoles. Significance was determined by 2way ANOVA, ***, p ≤ 0.001 and ns, not significant.

We observed significant recruitment of K63-ubiquitin chains to type II *Toxoplasma* PVs (Fig 1C). We tested the ability of the ubiquitin-binding proteins NDP52 and p62 to recognise type I and II *Toxoplasma* PVs in IFNγ-stimulated HUVEC. We found that both NDP52 and p62 localise to 20–30% of the type II PVs, 2.5h p.i. in IFNγ-stimulated cells (Fig 2A and 2B). Neither protein was recruited significantly to the PVs of type I parasites consistent with their lack of ubiquitin deposition (Fig 2A and 2B). Unlike the staining observed for ubiquitin, NDP52 and p62 staining was not continuous but occurred in patches around the PV, with NDP52 and p62 frequently occupying distinct domains (S4–S6 Figs). We extended our studies to super-resolution Structured Illumination Microscopy (SIM), which revealed that although p62 and NDP52 were most often found in separate microdomains, they did occasionally overlap (Fig 2C and S5 Fig).

Due to the ubiquitin-binding capacity of both p62 and NDP52, we assessed the recruitment kinetics of ubiquitin, p62 and NDP52 to the PV. A time-course of ubiquitin recruitment to the vacuole suggested that recruitment was rapid with 15% of type II PVs ubiquitin positive by 30 min post infection (p.i.), which increased to a plateau ~30% at 1h p.i. (Fig 2D). p62 was recruited to approximately the same levels as ubiquitin by 2h p.i., although with slower kinetics than ubiquitin. NDP52 recruitment also reached its plateau at 2h p.i., but accumulated on only half the number of PVs as ubiquitin and p62, with concurrent lower levels before 2h p.i. (Fig 2D). We thus hypothesised that ubiquitin is recruited first, with p62 and NDP52 being recruited subsequently, by virtue of their ubiquitin-binding domains. To test this hypothesis we inhibited ubiquitination by chemically blocking the E1 conjugating enzyme. This led to a significant decrease in ubiquitin-coated PVs, a corresponding significant decrease in p62 recruitment and a trend for less NDP52 around type II PVs (Fig 2E).

siRNA against p62 in HUVEC led to a loss of IFNγ-dependent restriction of type II *Toxoplasma*, indicating that p62 functions in the control of the intracellular parasite in an IFNγ-dependent manner (Fig 2F). Knock down of p62 was confirmed by immunoblotting (S7 Fig).

### *Toxoplasma* type I and II PVs recruit low level autophagy markers and are not cleared by autophagy

**Fig 3:**
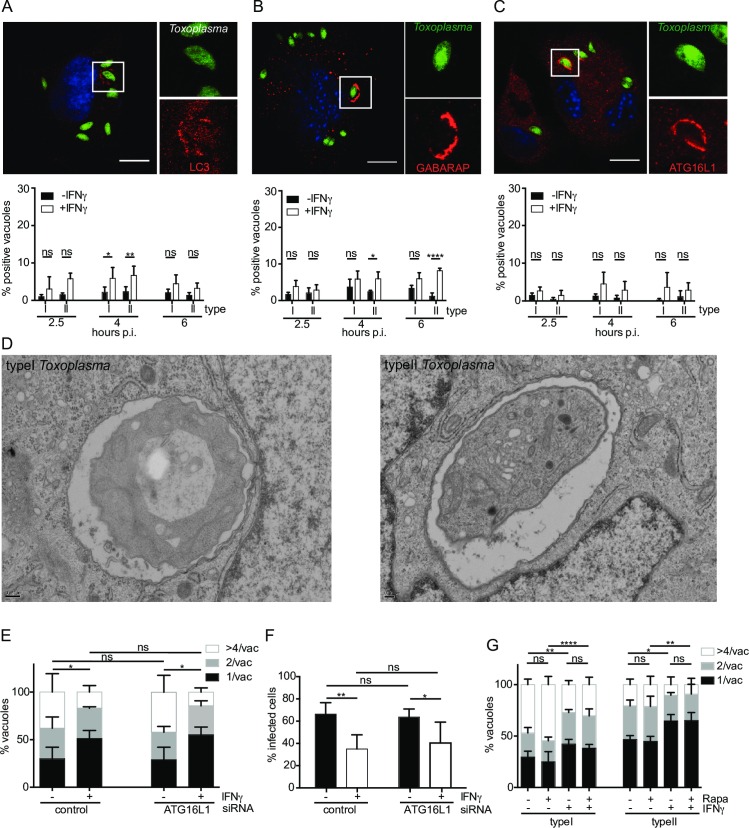
Autophagy does not control *Toxoplasma* replication in HUVEC. (A), (B) and (C) The autophagy proteins LC3B, GABARAP and Atg16L1 localise minimally to both type I and type II *Toxoplasma* in IFNγ-stimulated HUVEC. Confocal images were taken of HUVEC infected with type II *Toxoplasma* for 2.5, 4 and 6h, fixed and stained with LC3B, GABARAP or Atg16L1 antibodies. Representative images are shown. Scale bar 10μm. Quantitation of autophagy protein-positive PVs under the indicated conditions is shown. The mean of 3 experiments is shown. Significance was calculated using 2way ANOVA, *, p ≤ 0.05, **, p ≤ 0.01, **** p ≤ 0.0001 and ns, not significant. (D) Electron micrographs of type I and type II *Toxoplasma* in IFNγ-stimulated HUVEC exhibit no vacuolar disruption or obvious autophagosomal membranes. Ultrastructural analysis was performed in IFNγ-stimulated HUVEC 4h p.i.. Scale bar = 2μm. (E) Knock down of Atg16L1 does not impact IFNγ-mediated restriction of type I and type II *Toxoplasma* in HUVEC. HUVEC were knocked down for Atg16L1 by nucleofection, incubated for 24h, then stimulated or not with IFNγ for 24h before infecting with type II *Toxoplasma* for 18h. Results of ≥3 experiments are shown. Parasite numbers/vacuole were counted and significance determined using 2way ANOVA, *, p≤0.05 and ns, not significant. (F) HUVEC were knocked down for Atg16L1 by nucleofection of siRNA, incubated for 24h, then stimulated or not with IFNγ for 24h before infecting with type II *Toxoplasma* for 18h. Results of 5 experiments are shown. Percentage infected cells were determined in >100 cells by fluorescence microscopy and significance calculated by 2way ANOVA, **, p≤0.001, *, p≤0.05 and ns, not significant. (G) HUVEC were incubated for 24h with 50units/ml IFNγ and 100nM rapamycin, before washing and infecting with type I or type II *Toxoplasma* for 18h, when the cultures were fixed for microscopy. The number of parasites/vacuole was scored. The mean of 3–4 experiments is shown. Significance was calculated using 2way ANOVA, *, p≤0.05, **, p ≤ 0.01, ****, p ≤ 0.0001 and ns, not significant.

To assess whether type II PV was targeted for autophagic clearance, HUVEC left untreated, or primed with IFNγ were infected with type I or type II *Toxoplasma* and subsequently stained for LC3B, GABARAP or Atg16L1. A representative confocal image of each autophagy protein recruited to the type II PV is shown in Fig 3A, 3B and 3C. Although some IFNγ-dependent recruitment was observed, targeting of these autophagy molecules to PVs was found in ≤10% of vacuoles in IFNγ-primed HUVEC and did not exhibit parasite strain dependence up to 6h p.i. (Fig 3A, 3B and 3C). EM images were taken of both type I and type II *Toxoplasma* infections in IFNγ-stimulated HUVEC at 4h p.i. and showed no autophagosomes in the vicinity of the PVs, but rather close apposition of rough endoplasmic reticulum (Fig 3D and S8A Fig). This differs from the observations made for infection of mouse cells in which type II PVs are ruptured and targeted by autophagy [41–45]. To confirm our results, we stained PVs for galectin 8, a protein that has been shown to recognise exposed host glycans on damaged *Salmonella*-containing vacuoles and damaged lysosomes leading to the recruitment of NDP52 and autophagic destruction of the bacteria [46]. Galectin 8 was found to coat less than 7% of type II *Toxoplasma* in IFNγ-stimulated HUVEC while hardly recognising type I PVs or type II PVs in unstimulated cells (S8B Fig). Representative confocal microscopy images of galectin 8 recruitment to type II PVs are shown in S8C Fig at 2.5h p.i.. We thus concluded that the PV of neither type I nor type II *Toxoplasma* grossly breaks as is the case in IFNγ-stimulated murine cells. Of course one cannot exclude minor leakage of the PV, as potentially hinted at by the low level (~6%) galectin 8 staining we observed.

Despite our inability to find obvious autophagic membranes around *Toxoplasma* PVs by EM and the strain-independent low coating of PVs, we wanted to ascertain if autophagy played a functional role in type II parasite killing. We thus inhibited autophagy by knocking down Atg16L1 and confirmed levels of knock down by immunoblot (S9A Fig). Parasite replication was assessed after 18h and found to be not significantly different for the Atg16L1 knock down cells when compared with control siRNA-treated cells (Fig 3E). In order to establish the level of parasite clearance in Atg16L1 knock down compared to control siRNA-treated cells, the percentage infected cells at 18h post infection in HUVEC was determined. No significant difference was recorded from control cells (Fig 3F). As a further corroboration of the minimal effect of autophagy on the IFNγ-dependent control of *Toxoplasma* in HUVEC, we stimulated autophagy in HUVEC by pre-incubating cells for 24h with 100nM rapamycin concurrent with IFNγ-stimulation. Cells were then washed and infected with type I or II *Toxoplasma* for 18h and replication determined. Addition of rapamycin had no significant effect on parasite replication for either type I or type II *Toxoplasma* (Fig 3G). Confirmation of the increase in autophagy in HUVEC treated with rapamycin was made by immunoblotting treated cells for p62 and LC3B (S9B Fig), with bafilomycin A1 used post rapamycin treatment to allow visualisation of p62 and LC3B II which would otherwise be degraded by lysosomal enzymes. As expected for stimulation of autophagy, a decrease in p62 levels was observed on rapamycin treatment, as was an increase in conversion of LC3B I to LC3B II (S9B Fig). We conclude from these experiments that autophagy is neither the only nor the dominant route for elimination of type II parasites in HUVEC. This is despite the ubiquitin binding proteins p62 and NDP52 both having LC3 binding domains and having been associated with autophagy regulatory functions in bacterial infections in HeLa cells [27,30,47,48].

### Ubiquitinated type II *Toxoplasma* is destined for destruction by acidification in late endosomes/lysosomes

**Fig 4:**
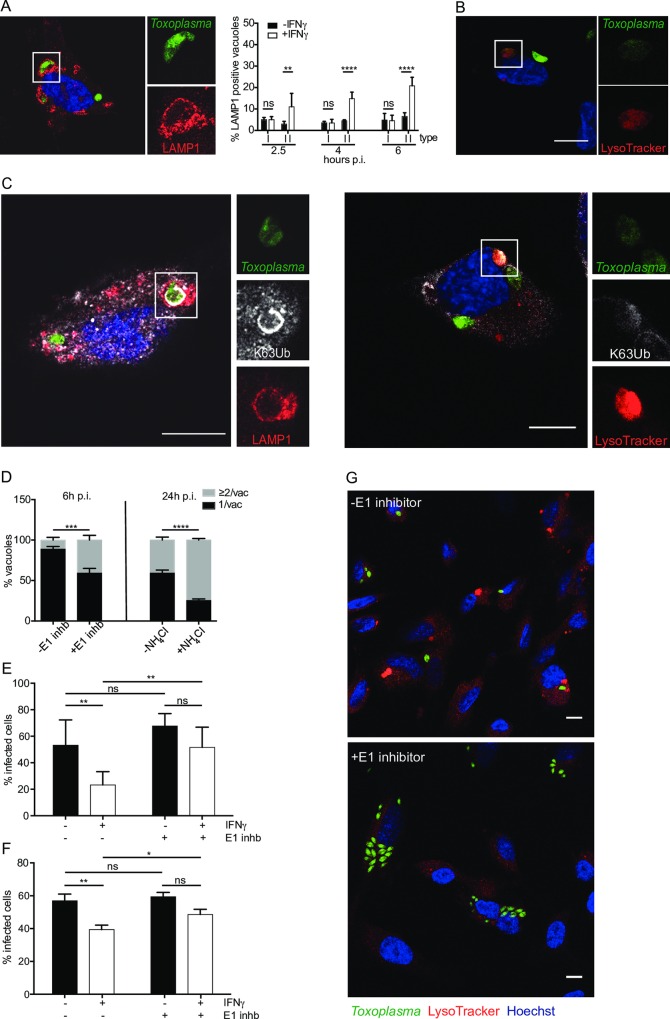
K63-ubiquitin-dependent endo-lysosomal acidification of *Toxoplasma* PV inhibits parasite replication. (A) Acidification of the PV is dependent on IFNγ and *Toxoplasma* virulence type. Type II *Toxoplasma* PV acidifies in IFNγ-stimulated HUVEC. Representative confocal image of infected cell stained with LAMP-1 is shown 4h p.i.. Scale bar = 10 μm. Quantitation of LAMP1 positive PVs under the indicated conditions is shown. The mean of ≥3 experiments is shown. Significance was determined using 2way ANOVA, **, p≤ 0.01, ****, p≤ 0.0001 and ns, not significant. (B) Representative confocal image of an infected cell stained with LysoTracker is shown 3h p.i.. Scale bar 10 μm. (C) Ubiquitinated type II *Toxoplasma* are destined for acidification in IFNγ-stimulated HUVEC. Representative confocal image showing K63-ubiquitin antibody co-staining with LAMP-1 antibody or LysoTracker, 4h p.i. Scale bar 10μm. (D) Inhibiting ubiquitination and acidification restores type II *Toxoplasma’s* ability to replicate in IFNγ-stimulated HUVEC. HUVEC were stimulated or not with IFNγ for 18h before being treated with 50μM UBEI-41 for 2h to inhibit host E1. Cells were then washed prior to infection with type II *Toxoplasma* for 6h. Replicating *Toxoplasma* counts with and without E1 inhibitor are shown, mean of ≥3 experiments. Significance was determined using 2way ANOVA, where *** indicates p ≤ 0.001. HUVECs were treated with 10mM NH_4_Cl 1h after infection with type II *Toxoplasma* to prevent acidification and infection continued in the presence of NH_4_Cl until 24h. Replicating *Toxoplasma* counts with and without NH_4_Cl are shown as parasites/vacuole, mean of 3 experimental replicates. Significance was determined using 2way ANOVA, ****, p ≤ 0.0001. (E) HUVEC were stimulated or not with IFNγ for 18h before being treated with 50μM UBEI-41 for 2h to inhibit host E1. Cells were then washed prior to infection with type II *Toxoplasma* for 18h. Percentage infected cells were counted in >100 cells. The mean of 4 experiments is shown. Significance was determined using 2way ANOVA, **, p ≤ 0.01 and ns, not significant. (F) HUVEC were stimulated or not with IFNγ for 18h before being treated with 50μM UBEI-41 for 2h to inhibit host E1. Cells were then washed prior to infection with type II *Toxoplasma* for 18h. Infected cells were prepared and fixed for FACS and cells containing fluorescent parasites scored as a measure parasite survival. Results are expressed as percentage infected cells. A representative of 3 experiments is shown, each performed in triplicate. Significance was determined using unpaired student t test, **, p ≤ 0.01, *, p ≤ 0.05 and ns, not significant. (G) Inhibiting ubiquitination decreases the presence of LysoTracker-positive structures in IFNγ-stimulated HUVEC infected with type II *Toxoplasma* 6h p.i.. Confocal image of infected cells stained with LysoTracker in the presence or absence of the E1 inhibitor UBEI-41. Scale bar 10 μm.

We next assessed which host cellular destruction pathway plays an important role in eliminating type II *Toxoplasma* from IFNγ-stimulated HUVEC. We postulated that the type II *Toxoplasma* PV fuses with the cellular endocytic pathway rather than routes the parasite to destruction by autophagy. To determine whether *Toxoplasma* PVs intersected the endo-lysosomal system, we performed LAMP1 staining at 2.5h, 4h and 6h p.i. with type I and type II *Toxoplasma*. We found that 10–20% of type II *Toxoplasma* PVs in IFNγ-stimulated HUVEC were LAMP-1 positive throughout all time points, while less than 7% LAMP-1 localisation to PVs was observed in cells infected with type I *Toxoplasma* or type II *Toxoplasma* in the absence of IFNγ (Fig 4A). Additionally, we observed the accumulation of the late endosome protein Rab7 to the PV at 2.5h p.i., consistent with an endo-lysosomal route for destruction of type II *Toxoplasma* in IFNγ-stimulated HUVEC (S10A and S10B Fig). The parasite in S10B Fig has lost fluorescence due to acid-lability of eGFP or parasite degradation, with only the Hoechst staining visible. As an additional measure for vacuolar acidification, we added LysoTracker red to cells infected with type II parasites at 2h p.i. and incubated the cultures for a further 1 hour before fixation and microscopy analysis. LysoTracker staining was observed over parasites that appeared to be ‘sick’ or dying (Fig 4B).

We noted that it was indeed the vacuoles coated with ubiquitin that frequently contained either “unhealthy” parasites or DNA (Hoechst) positive parasites that had lost their fluorescence. This could be due to parasite death and subsequent loss of the parasite cytoplasmic fluorescence, or acidification of the vacuole leading to loss of acid-labile eGFP fluorescence. Thus we examined if ubiquitin positive PVs correlate with ones that acidify. We found that a fraction of PVs that contain K63-linked ubiquitin, are also positive for LAMP1 and LysoTracker (Fig 4C and S11 and S12 Figs). EM images at 4h p.i., revealed a set of type II parasites that are being digested in an endosome/lysosome compartment (S13 Fig). Approximately 45 percent of the EM images of type II infected IFNγ-stimulated HUVEC showed these enlarged lysosome structures and it was noted that more of these structures were present in type II compared with type I infected cells. Note that at 2.5h p.i not all ubiquitin-positive vacuoles stain with LysoTracker or are LAMP-1 positive (Figs 2D and 4A). This suggests that ubiquitination precedes acidification of the vacuole.

What is the fate of ubiquitin-coated type II *Toxoplasma* PVs that acquire cellular markers of acidification? In order to answer this, we blocked host-driven ubiquitination with the E1 inhibitor UBEI-41 or neutralised lysosomes with NH_4_Cl. Both treatments markedly rescued the ability of type II *Toxoplasma* to replicate in the presence of IFNγ (Fig 4D). Furthermore, parasite clearance, as measured by the percentage of infected cells at 18h p.i., demonstrated that in the presence of IFNγ, parasites showed significantly better survival when cells were pre-treated with E1 inhibitor (Fig 4E). As an additional measure of parasite clearance, we used a FACS-based assay to count the percentage of infected cells. Again, in the presence of IFNγ, the cells pre-treated with the E1 inhibitor showed increased *Toxoplasma* viability compared with untreated cells (Fig 4F). A representative image of HUVECs infected with type II *Toxoplasma* and either treated or not with the ubiquitination inhibitor shows more parasites per field of view in treated cells (Fig 4G). Additionally, it is noticeable that general LysoTracker staining denoting acid compartments are reduced in cells blocked in their ubiquitination capacity.

### PV ubiquitination in HeLa cells drives distinct non-acidification dependent growth restriction pathway of *Toxoplasma*

**Fig 5:**
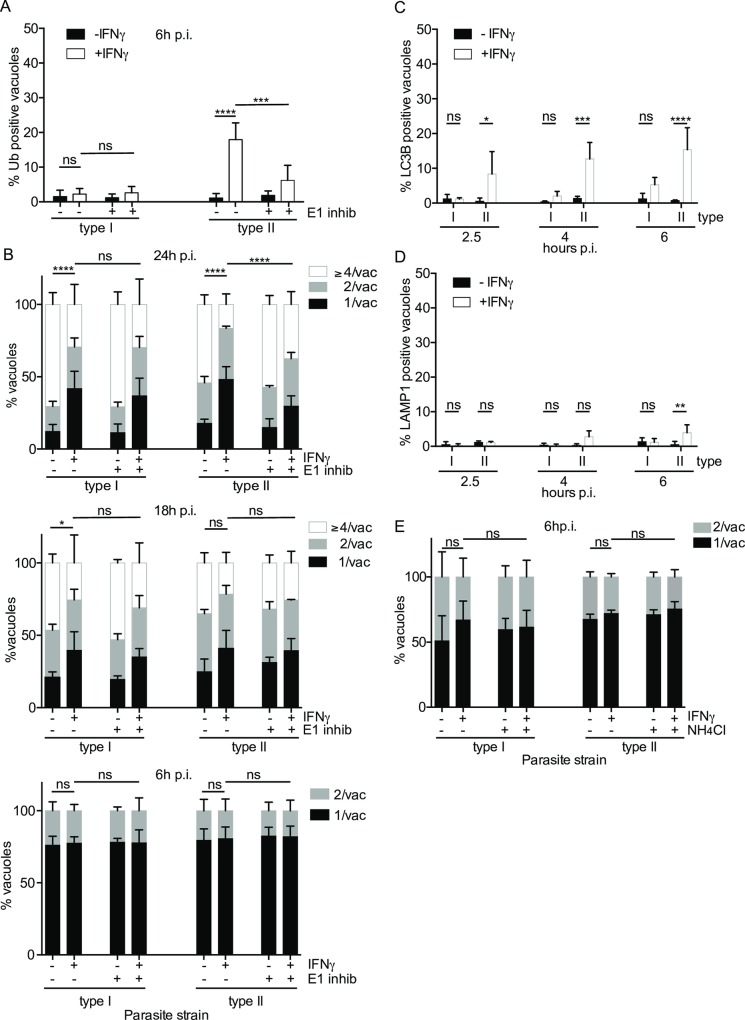
HeLa cells retard growth of *Toxoplasma* by an IFNγ and ubiquitin-dependent but acidification-independent mechanism. (A) Ubiquitin is recruited to type II *Toxoplasma* vacuoles in IFNγ-stimulated HeLa cells. Inhibiting ubiquitination with the E1 inhibitor UBEI-41 reduces recruitment of ubiquitin to type II vacuoles at 6h p.i.. Counts are expressed as % positive vacuoles and the mean of 3 experiments is shown. Significance was determined using 2way ANOVA, ***, p ≤ 0.001, ****, p ≤ 0.0001 and ns, not significant. (B) Inhibiting ubiquitination increased the ability of type II *Toxoplasma* to replicate in IFNγ-stimulated HeLa at 24h and 18h p.i., but no difference in replication was observed at 6h. HeLa were pre-treated with 50μm UBEI-41 to inhibit ubiquitination prior to infecting with type I or type II *Toxoplasma* for 24, 18 and 6h. Replicating *Toxoplasma* counts with and without inhibition of ubiquitination are shown, mean of ≥4 experiments are shown Significance was determined using 2way ANOVA where * indicates p ≤ 0.05, **** indicates p ≤ 0.0001 and ns not significant. (C) LC3B is recruited to the type II vacuole. HeLa cells stimulated or not with IFNγ for 18h were infected with type I or type II parasites for the indicated times. Recruitment of LC3B was monitored by antibody staining. Graphs indicating the percentage of positive vacuoles are shown for 3 experiments. Significance was determined using 2way ANOVA, *, p ≤ 0.05, ***, p ≤ 0.001, ****, p ≤ 0.0001 and ns, not significant. (D) The type II *Toxoplasma* vacuole in IFNγ-stimulated HeLa cells does not acquire lysosomal markers and does not acidify. HeLa cells were infected with type I or type II *Toxoplasma* for the indicated times. LAMP1 staining was recorded and expressed as % positive vacuoles for 3 experiments. Significance was determined using 2way ANOVA, **, p ≤ 0.01 and ns, not significant. (E) Inhibiting acidification has no effect on the ability of type II *Toxoplasma* to replicate in IFNγ-stimulated HeLa, 6h p.i.. HeLa cells were treated with 10mM NH_4_Cl 1h after infection to prevent acidification and infection continued until 6h. Replicating *Toxoplasma* counts with and without NH_4_Cl are shown, mean of ≥3 experiments are shown. Significance was determined using 2way ANOVA, ns = not significant.

It has been reported that IFNγ-dependent ubiquitination of type II and III *Toxoplasma* PVs route the parasite for growth stunting by non-canonical autophagy [26]. Importantly, this pathway does not rely on acidification of the PV. In this previous study, the markers of host defence on the parasite PVs in HeLa were determined at 6h p.i. (ubiquitin, p62, NDP52, LC3B and LAMP1), while the restrictive effect on parasite replication was assessed and found to have an effect at 24h p.i.. For the present study in HUVEC, we have conducted time courses from 2–6h p.i. for the markers of host defence, while also finding that in a window of 6–24h p.i., restriction of the parasite’s replicative capacity was dependent on the ubiquitin host defence system. We first confirmed that ubiquitin is indeed recruited to the type II PV of *Toxoplasma* in HeLa cells in an IFNγ-dependent manner (Fig 5A). Additionally, inhibiting the E1 with UBEI-41 significantly reduced the ubiquitin coating (Fig 5A). Next, we ascertained that at 24h p.i., replication of both type I and II parasites was restricted in HeLa cells by IFNγ (Fig 5B). Inhibiting host-driven ubiquitination significantly rescued the replicative capacity of type II parasites, while having no effect on type I parasites (Fig 5B). Interestingly, when assessing the IFNγ-dependent restrictive capacity of HeLa cells on type I and II *Toxoplasma* earlier than 24h p.i., namely at 18h p.i. and 6h p.i., we only detected a slight restriction of both parasite types at 18h and no restriction at 6h post-infection (Fig 5B). Inhibiting host ubiquitination also did not rescue the slight IFNγ-driven restriction of type II *Toxoplasma* replication at 18h p.i. (Fig 5B). These earlier time points of host restriction were not previously assessed in HeLa cells, however, they present first evidence that the pathways of IFNγ-mediated ubiquitin-driven restriction of type II *Toxoplasma* are indeed different in primary-like HUVEC versus HeLa cells.

While HUVEC did not exhibit a distinct IFNγ-dependent, *Toxoplasma* strain-dependent coating of the PV with the autophagy markers LC3B, GABARAP and Atg16L1, HeLa cells were shown to facilitate the recruitment of LC3B to type II *Toxoplasma* in a IFNγ-dependent fashion [26]. We confirmed that indeed at 6h p.i. in IFNγ-stimulated HeLa cells, LC3B was found on type II *Toxoplasma* PVs at 15–20% (Fig 5C). To extend this finding, we analysed LC3B recruitment to the PV earlier than 6h p.i. and could detect it on type II *Toxoplasma* PVs at 2.5h and 4h p.i. (Fig 5C). This again highlights that primary-like HUVEC and HeLa cells use different routes to inhibit type II *Toxoplasma* after priming with IFNγ.

A major hallmark of IFNγ-driven ubiquitin-mediated elimination of type II *Toxoplasma* in HUVEC that we have identified in this study is the acidification of the PV and subsequent destruction of the parasite. Ubiquitin-targeted PVs in HeLa cells were found not to acidify, but instead restrict type II *Toxoplasma* through growth stunting. We confirmed that indeed in HeLa cells, LAMP1 is not recruited to *Toxoplasma* PVs at 2.5h, 4h and only marginally (~5%) to type II at 6h p.i. (Fig 5D). Unlike HUVEC and in agreement with this finding, the neutralisation of lysosomes with NH_4_Cl does not enhance *Toxoplasma* replication in HeLa cells (Fig 5E). Thus we concluded that HeLa cells do not route type II *Toxoplasma* into an acidification-dependent restriction pathway as we observe in HUVEC (S1 Table).

## Discussion

IFNγ is the major cytokine controlling both acute and chronic phase *Toxoplasma* infection *in vivo*. In this report, we define ubiquitin-driven vacuolar fusion with the endo-lysosomal system as a novel host cell-autonomous restriction mechanism of type II, but not type I *Toxoplasma* in IFNγ-primed human primary-like endothelial cells. IFNγ can restrict both type I and type II *Toxoplasma*, but is more effective against type II parasites measured at 24h p.i. and significant from 6h p.i (Fig 1A). Other mechanisms that restrict type I and type II parasites in endothelial cells must therefore exist. Induction of indoleamine dioxygenase (IDO1) by IFNγ appears not to be relevant for type II *Toxoplasma* restriction in HUVEC, since supplementation of cultures with tryptophan has no effect on replication (S14 Fig).

We have shown that in IFNγ-primed HUVEC type II and not type I *Toxoplasma* PVs undergo ubiquitination and subsequent acidification leading to parasite killing. We set out to determine the functional consequences of inhibiting either PV ubiquitination or acidification on parasite replication. To assess the role of ubiquitination we used UBEI-41, an inhibitor of the cellular E1 ubiquitin-activating enzyme UBA1. When IFNγ-primed HUVEC infected with type II *Toxoplasma* were treated with UBEI-41, a reduction in IFNγ-dependent vacuolar ubiquitination was observed and the effect of IFNγ on ubiquitin coating of the PV became insignificant (Fig 2E). Concordantly, decreased PV ubiquitination rescued the ability of the type II *Toxoplasma* to replicate in IFNγ-primed HUVEC (Fig 4D) and led to increased parasite survival (Fig 4E and 4F). Consistent with our observation that ubiquitination of PVs precedes acidification, inhibiting ubiquitination with UBEI-41 led to less acidification of type II PVs in HUVEC, and significantly more parasites (Fig 4G). This prompted us to directly assess the role of acidification in parasite killing. For this, vacuolar acidification was buffered through the addition of NH_4_Cl. As observed upon inhibition of ubiquitination, inhibition of vacuole acidification rescued the type II parasite’s ability to survive and grow in IFNγ-stimulated HUVEC (Fig 4D). These results indicate that ubiquitination and acidification of type II *Toxoplasma* vacuoles is required for parasite death in IFNγ-stimulated HUVEC. They further demonstrate that ubiquitination is a prerequisite for PV endosome/lysosome fusion and subsequent PV acidification.

Contrary to mouse non-haematopoetic cells, the type II PV in IFNγ-stimulated human cells does not exhibit ruffling and major breakage up to 4h p.i. (Fig 3D, S8A Fig and [26]). This is likely due to the absence of IFNγ-inducible p47 GTPases (IRGs) from the human genome, as it is these proteins that are crucial for mediating vacuolar breakage [44]. It is also the two murine IFNγ-sensitive IRGs Irgm1/3 that are responsible for mediating recruitment of ubiquitin, p62 and the E3 ligase Traf6 to type II *Toxoplasma* PVs [49]. Whether the non-IFNγ-inducible human IRGM plays a role in the vacuolar acidification pathway or other human host resistance mechanisms remains to be investigated. The K63-linked, ubiquitin-mediated endo-lysosomal fusion and acidic killing mechanism we observe in human endothelial cells is however strictly dependent on IFNγ. This leads us to speculate that the determining factors for the new host defence mechanism are IFNγ-inducible and may possibly be one or more E3 ubiquitin ligases.

It is intriguing to speculate however that there may be minor disruptions of the PV membrane of type II *Toxoplasma*, as we do observe almost 7% of these PVs in IFNγ-stimulated cells to stain positive for galectin 8 albeit this being only a trend with IFNγ and not statistically significant (S8B Fig). Galectin 8 has previously been used as a marker for broken *Salmonella* vacuoles by detecting host vacuolar glycans exposed to the cytoplasm [46]. The significance of minor PVM damage in this scenario remains to be investigated. Furthermore, galectin 8 has been reported to activate antibacterial autophagy by recruiting NDP52 to broken pathogen vacuoles [46], but from our observations, the percentage of NDP52 positive vacuoles is more than double the number of galectin 8 positive vacuoles, implying that NDP52 has a different role in type II *Toxoplasma* infections of HUVEC.

Autophagic clearance initiated by ubiquitination and subsequent lysosomal fusion and killing of intracellular bacteria is a well-described phenomenon. Bacterial pathogens are recognised constitutively in human cells, while we find that type II *Toxoplasma* is only targeted by ubiquitin in IFNγ-stimulated cells. Endothelial cells as well as fibroblasts have previously been determined not to deploy autophagy to kill *Toxoplasma* and it had remained unclear how the parasite is eliminated [17,25]. In line with these previous studies we also do not find autophagy to be the major host resistance pathway, as knock down or induction of autophagy had absolutely no effect on the parasite’s replicative capacity as well as the percent infected host cells. Additionally, we could demonstrate that all three major autophagy markers LC3B, GABARAP and Atg16L1 were recruited to PVs in lower amounts, but without a strain-dependent pattern.

However, as with bacterial clearance, NDP52 and p62 are also present at the PV of type II *Toxoplasma*, and interestingly in distinct microdomains. This may be due to interaction with different ubiquitin linkage partners. p62 is known to bind both K48 and K63 linked ubiquitin, but with a preference for K63 linkages [50–52]. NDP52 has been reported to occupy domains on cytosolic salmonella that are distinct from p62 but overlapping with optineurin which was shown to bind linear polyubiquitin chains, suggesting a likely binding of NDP52 to linear ubiquitin [28]. Interestingly, we observe broadly similar percentages of K63 ubiquitin and p62 positive vacuoles (25–30%) and equally, levels of M1 linear ubiquitin mirror the percentage NDP52 staining (15–20%) on type II vacuoles. The IFNγ-driven restriction of type II *Toxoplasma* is removed when p62 is knocked down in HUVEC, implying that this ubiquitin binding protein is likely mediating its effect via a signalling mechanism rather than through autophagy. It is possible that NDP52 functions in a similar manner since the only Atg8 protein it is able to bind is LC3C [53] and we could not detect LC3C binding to vacuoles of type I or type II *Toxoplasma* in IFNγ-stimulated HUVEC, indicating the lack of an autophagic role for NDP52. Future work will seek to detail the autophagy-independent function of these host proteins at the PV membrane.

Contrasting our study in human primary-like endothelial cells, another study has found that in IFNγ-stimulated epithelial HeLa cells ubiquitination of type II and III and not type I *Toxoplasma* vacuoles leads to parasite growth stunting via non-canonical autophagy without acidification [26]. We have confirmed the findings that ubiquitination of the PV in HeLa cells is also *Toxoplasma* strain-specific and IFNγ-driven (Fig 5A). Moreover, we confirmed that the autophagy marker LC3B is recruited to these PVs (Fig 5C), while little acidification is detected (Fig 5D). We have summarised our findings in HUVEC and HeLa compared to the previous study in HeLa in S1 Table. To extend these observations in HeLa cells and to compare the kinetics of *Toxoplasma* restriction to HUVEC used in this study, we have additionally performed *Toxoplasma* replication assays in HeLa cells at 6, 18 and 24h p.i., with and without the inhibition of ubiquitination. We could clearly show that while in HUVEC restriction of type II *Toxoplasma* was already observable at 6h p.i., in HeLa cells this was not apparent until 24h p.i. (Fig 1A versus 5B). Nevertheless, for both cell types, the type II *Toxoplasma* restriction was dependent on ubiquitination and could be reduced by inhibiting the host ubiquitin pathway (Figs 4D, 4E and 4F versus 5B). We additionally neutralised lysosomes with NH_4_Cl in HeLa cells and could demonstrate that concurrent with the lack of acidic markers and in contrast to our findings in HUVEC, this did not rescue *Toxoplasma* replication in HeLa cells (Figs 4D versus 5E). We concluded that the previously described host restriction pathway in HeLa cells is, as the authors describe, a “growth stunting” of the type II parasites via an autophagy pathway. This likely explains why the impact on parasite replication takes much longer to be apparent in HeLa than in HUVEC. It remains to be determined which ubiquitin linkage(s) HeLa cells deploy to the PV and if and how the parasite is eventually killed and eliminated. The two different human cell types deploy the same initial defence molecules to similar quantities (ubiquitin, p62, NDP52) and then diverge in their ultimate strategy on how to destroy the parasite. While HUVEC present with a lysosomal acidification of the vacuole, HeLa deploy a higher quantity of autophagy markers to the PV and stunt the growth of the parasite. We pursued the lack of significant autophagy in HUVEC by assessing recruitment of additional Atg8 proteins, LC3C and GABARAP. We did not observe any staining with LC3C. The GABARAP antibody we employed detects all GABARAP subforms and amounts to about 5% on the PVs. Thus, if LC3B and GABARAP decorate distinct vacuoles, the sum of their recruitment would be around 10%, still far less than observed in HeLa. Accordingly, we still believe the main host effector mechanism that restricts *Toxoplasma* downstream of Ubiquitin/p62/NDP52 in HUVEC and HeLa are different. However, given that we find residual levels of LAMP1 on HeLa PVs and LC3B, GABARAP and Atg16L1 on HUVEC PVs, with some IFNγ-dependent significance, it is conceivable that the ubiquitin downstream defence mechanisms in these two cell types are simply present at varying quantities. Hence, in HUVEC there may be a basal IFNγ-dependent autophagy in both type I and II *Toxoplasma*, but insufficient to clear the parasites effectively, as we were unable to restore parasite viability by Atg16L1 knock down. In this context it is important to note that HeLa cells have been shown to have an unusually high level of basal autophagy [55,56].

**Fig 6:**
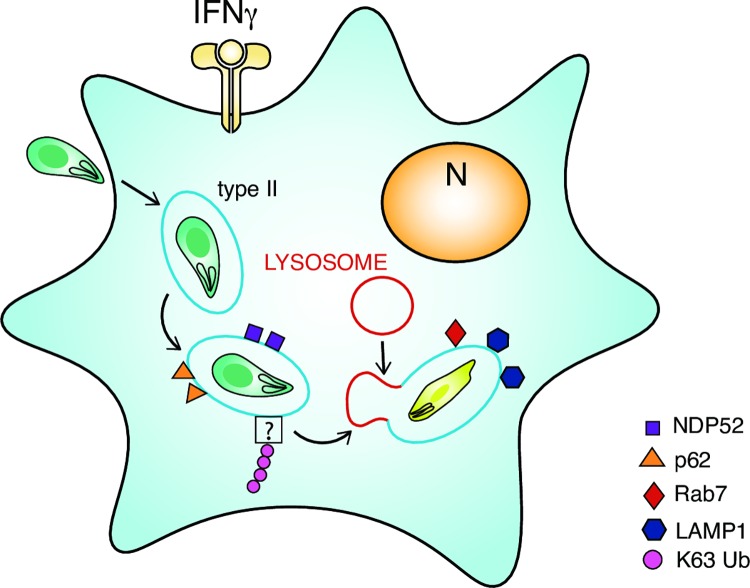
Model for K63 ubiquitin-dependent endolysosomal destruction of type II *Toxoplasma* in IFNγ-stimulated HUVEC. Upon entry into IFNγ-stimulated HUVEC, type II *Toxoplasma* (green) enveloped within their PV (blue line) are recognised by host immune effectors (K63Ub, p62, NDP52, LAMP1, Rab7) leading to lysosomal fusion and subsequent destruction of the parasite.

In contrast, HUVEC ubiquitinate and send type II *Toxoplasma* to acidic destruction by means of the PV becoming endo-lysosomal. This process is much faster and results in parasite death and degradation. K63-ubiquitin recognition renders the *Toxoplasma* vacuole fusogenic with the endocytic pathway and to our knowledge presents the first physiologically relevant observation of *Toxoplasma* vacuolar acidification. A model proposing our observations in HUVEC is made in Fig 6. An important question for the future is, does ubiquitin-driven acidification only exist in primary-like endothelial cells or is it also deployed in human macrophages? Our discovery of a human host acidification dependent destruction pathway opens the door to determining the parasitic virulence factors used by *Toxoplasma* to evade recognition in human cells. These combined efforts might uncover novel host and pathogen targets for the development of anti-*Toxoplasma* compounds.

## Methods

### Cell culture

Human Umbilical Vein Endothelial cells, HUVECs, (Promocell C12203), were maintained in M199 medium (Life Technologies) supplemented with 30μg/ml Endothelial cell growth supplement (ECGS) (Upstate 02–102), 10units/ml heparin (Sigma H-3149) and 20% FBS (Life Technologies). Cells were grown on plates, pre-coated with 1% (w/v) porcine gelatin (Sigma G1890) and cultured at 37°C in 5% CO_2_. HUVEC were not used beyond passage 6. HeLa (ECACC, Sigma) and human foreskin fibroblasts, HFFs (ATCC), were cultured in DMEM with GlutaMAX (Life Technologies) supplemented with 10% FBS (Life Technologies), at 37°C in 5% CO_2_.

### Interferon stimulation of cells

HUVEC and HeLa were stimulated for 18–24h in complete medium at 37°C with addition of 50units/ml human IFNγ (R&D Systems).

### Parasite culture

*Toxoplasma gondii* expressing luciferase/eGFP or tdTomato (Prugniaud type II, CEP type III and RH type I) were maintained *in vitro* by serial passage on monolayers of HFF cells, cultured in DMEM with GlutaMAX (Life Technologies) supplemented with 1% FBS (Life Technologies), at 37°C in 5% CO_2_. Parasite virulence mutants Prugniaud ROP16_I_ and CEP ROP18_I_ were a gift from J P Saeij, MIT, MA.

### *Toxoplasma* infection

*Toxoplasma* were prepared from freshly 25G syringe-lysed HFF cultures in 1% FBS, adding to experimental cells at a multiplicity of infection (MOI) of 2–5:1 for type II and III strains and 0.5–1:1 for type I strain. The cell cultures with added *Toxoplasma* were then centrifuged at 1000rpm for 5 min to synchronise the infection, prior to culturing at 37°C, 5% CO_2_ for the required time.

### Reagents and inhibitors

Ubiquitin E1 inhibitor UBEI-41 was from Biogenova. Cells were pre-treated with 50μM UBEI-41 for 2h and washed 3 times in medium prior to infection. NH_4_Cl and L-tryptophan were from Sigma-Aldrich.

Acidification of parasite vacuoles was monitored using LysoTracker-red DND99 Molecular Probes (L7528 ThermoFisher); the dye has good retention post fixation with aldehydes and so was suitable for fixed immunofluorescence. LysoTracker-red was added at a concentration of 50nM, 1 hour prior to fixation.

Induction of autophagy was achieved using rapamycin (Sigma, R8781) and inhibition of autophagy by bafilomycin A1 (Sigma, B1793). Rapamycin was added to cultures at a concentration of 100nM 24h prior to infection and the cells washed in 3x in medium before adding parasites. Bafilomycin A1 was added to cultures at a concentration of 400nM after rapamycin treatment and 2h prior to lysing cells.

### siRNA

siRNA for Atg16L1 (mix of 3 Silencer Select Ambion #4392420: s30069, s30070, s30071) and siRNA control (AM4635) were from ThermoFisher. Sequences for p62 siRNA (p62: GCAUUGAAGUUGAUAUCGAU[dT][dT], p62_as: AUCGAUAUCAACUUCAAUGC[dT][dT] [29] were synthesised by Sigma. Cells were transfected with siRNAs (50–100pmol) by nucleofection, Lonza (HUVEC old formulation VPB-1492) for 24h then IFNγ-stimulated and used after a further 24h. Efficiency of knock down was monitored by immunoblotting lysates of transfected cells.

### Antibodies

Rabbit polyclonal antibodies were α-p62 (Cliniscience, PM045), α-NDP52 (AbCam, ab68588), α-LC3B (AbCam ab48394), α-GABARAP (Abgent, AP1821a). Rabbit monoclonal antibodies were α-LC3B (Cell Signalling, 3868P for immunoblotting), α-ubiquitin Lys-63 specific, Apu3 (Merck Millipore, 05–1308), α-ubiquitin Lys-48 specific, Apu2 (Merck Millipore, 05–1307), α-ubiquitin M1 linear-specific, 1E3 (Merck Millipore, 199), α-Atg16L1 D6D5 (Cell Signalling, 8089), α-Rab7 D95F2 (Cell Signalling, 9367). Mouse monoclonal antibodies were α-ubiquitin FK2 (Enzo Life Sciences, PW8810), α-p62 (abcam, 56416), α-LAMP-1 H4A3 (Abcam ab25630), α-beta actin (Sigma, A2228). Goat polyclonal antibodies were α-galectin 8 (R&D systems, AF1305). Secondary antibodies used were Alexa Fluor 488-, or Alexa Fluor 568-, Alexa Fluor 647-conjugated chicken/goat α-rabbit, chicken/goat α-mouse or donkey α-goat (Molecular Probes).

### Immunoblotting

Adherent cells were washed 2x with ice cold PBS before scraping in ice cold cell lysis buffer (25 mM Tris HCl pH 7.4, 5mM MgCl_2_, 150mM NaCl, 1% Triton X-100 with protease inhibitor cocktail III, EDTA free; Calbiochem). Lysates were run on SDS PAGE 10μg/lane, blotted and blocked overnight in 5% nonfat milk, 0.05% Tween 20 in PBS with 0.02% sodium azide. Blots were incubated for 1h RT with primary antibody diluted in PBS with 0.05% Tween 20 (PBS Tween) and 1% milk. After 3x washes in PBS Tween, blots were incubated in second antibody-HRP 1h RT washed 3x PBS Tween and developed using Immobilon Western Chemiluminescent HRP substrate (Merck Millipore, WBKLS0500).

### Microscopy

#### Fixed Immunofluorescence microscopy

Cells were plated on coverslips (12mm, ♯1.5, ThermoFisher) (coated with 1% gelatin) in a 24-well plate and cultured, IFNγ–stimulated and infected with *Toxoplasma* as described above. The cells mounted on coverslips were prepared for imaging as detailed in S1 Appendix. Primary and secondary antibody incubations were carried out sequentially followed by washes of 3x 1ml PGAS, 2x 1ml PBS, and then by 1ml PBS containing 1μg/ml Hoechst 33342 (Life Technologies). Finally samples were washed twice in dH_2_O prior to mounting on glass slides with Mowiol 4–88 (Polysciences Inc.). Staining protocol is detailed further in S1 Appendix. Slides were viewed on a Zeiss Axioplan II Epifluorescence microscope using x100 objective, imaged with an AxioCam HRC camera and analysed with Axiovision 4.8 software or on an SP5-invert Confocal microscope using x100 objective and analysed using LAS-AF software.

#### Superresolution Structured Illumination Microscopy (SR-SIM)

SR-SIM imaging was performed using Plan-Apochromat 63x/1.4 oil DIC M27 objective, in an Elyra PS.1 microscope (Zeiss). Images were acquired using details described in S1 Appendix. 3D Images (z-slice with 0.25 μm interval) were acquired using a sCMOS camera (Andor) and processed using the ZEN software (2012, version 8.1.6.484, Zeiss). For channel alignment, a multicoloured bead slide was imaged using the same image acquisition settings and used for the alignment of the different channels.

#### Transmission electron microscopy

HUVEC were induced with IFN**γ** for 24h and infected with type II *Toxoplasma* for 4h. Cells were washed 3x in PBS before 0.05% tryspin-EDTA treatment. Samples were prepared according to details further described in S1 Appendix. Samples were observed with a JEOL 1200 EX transmission electron microscope (JEOL, Tokyo, Japan) equipped with an Orius 1000 CCD camera (Gatan, Pleasanton, CA, USA).

#### *Toxoplasma* viability assays and recruitment of markers to PV using microscopy

Parasite viability/replication was determined by counting the number of parasites per vacuole at the times specified for >100 vacuoles. Additionally, the number of infected cells was counted in >100 cells and expressed as % infected cells. Recruitment of markers to *Toxoplasma* PVs was assessed by counting the number of positive vacuoles in >100 vacuoles.

### *Toxoplasma* viability assay using flow cytometry

Infected cells were washed twice in PBS and lifted with 2X trypsin (Life Technologies), before quenching in DMEM 10%FBS. The cell pellet was washed in PBS before staining with a fixable live/dead near infrared stain (Thermofisher) at 1/2000 in PBS for 20min on ice. PBS was added to quench the reaction before the suspension was centrifuged at 1250 rpm for 5 min at 4°C. The cell pellet was then fixed in 3% paraformaldehyde in PBS, for 20min on ice. PBS was added to quench the reaction before centrifugation at 1250 rpm for 5min at 4°C. The cells were resuspended in PBS 1%BSA 2.5mM EDTA and analyzed on a BD LSR-II FACS. Results were analyzed using FlowJo V.10.1 software.

### Data handling, statistical measurements and evaluation

Numerical data was plotted using Graph Pad Prism and presented with error bars as standard deviation. Significance of results was determined by 2way ANOVA or unpaired t-test.

## Supporting Information

S1 Fig Ubiquitin surrounds the type II *Toxoplasma* PV in a homogenous coat. Movie of consecutive confocal 0.4μm separation z stacks of the type II PV, in IFN**γ**-stimulated HUVEC, stained with total ubiquitin antibody. *Toxoplasma* (green), ubiquitin (red), Hoechst (blue).
(MOV)

S2 Fig Ubiquitin surrounds the type II *Toxoplasma* PV in a homogenous coat. Movie of consecutive confocal 0.4μm separation z stacks of the type II PV, in IFN**γ**-stimulated HUVEC, stained with total ubiquitin antibody. *Toxoplasma* (green), ubiquitin (red), Hoechst (blue).
(MOV)

S3 Fig Virulence factors known to act in infections of murine cells are irrelevant for *Toxoplasma* infection of human cells. HUVEC stimulated or not with 50units/ml IFNγ type II (Pru) and type III (CEP) virulence mutants CEP-ROP18_I_ and Pru-ROP16_I_ and their controls for 2.5h before fixation for fluorescence microscopy. Ubiquitin positive vacuoles were counted in >100 vacuoles. The mean of 3 experiments is shown. Significance was determined by 2way ANOVA, ns, not significant.
(TIF)

S4 Fig p62 and NDP52 surround the type II *Toxoplasma* vacuole in overlapping microdomains. Movie of consecutive 0.4μm confocal z stacks of the type II PV, in IFN**γ**-stimulated HUVEC, co-stained with p62 (green) and NDP52 (magenta), antibodies is shown, with *Toxoplasma* (red), Hoechst (blue).
(MOV)

S5 Fig p62 and NDP52 surround the type II *Toxoplasma* vacuole in overlapping microdomains. Movie of a 3D reconstruction of a z stack of one representative Superresolution Structured Illumination Microscopy image is shown of type II PV. NDP52 (red), p62 (green), *Toxoplasma* (white) and Hoechst (blue) are shown. Scale bar 2μm.
(MOV)

S6 Fig Ubiquitin coats the type II vacuole whereas p62 is present in microdomains. Superresolution Structured Illumination Microscopy image of ubiquitin and p62 co-staining of type II PV. Total ubiquitin (red), p62 (green), *Toxoplasma* (white) and Hoechst (blue) are shown. Scale bar 2μm
(TIF)

S7 Fig p62 is knocked down in HUVEC by siRNA. Immunoblot showing lysates of HUVEC cells treated with siRNA control and p62 and probed with antibody to p62. Loading control is shown with antibody to β-actin.
(TIF)

S8 Fig Electron micrographs showing that both type I and type II *Toxoplasma* in IFNγ-stimulated HUVEC exhibit no vacuolar disruption. (A) Additional electron micrographs all demonstrate that the PVs containing type I or type II *Toxoplasma* do not break in IFNγ-stimulated HUVEC. Arrows indicate rough endoplasmic reticulum closely apposed to the vacuoles of both type I and type II parasites, enlarged view in boxes. Scale bar = 0.2μm (or 0.5μm centre left and top right). (B) HUVEC stimulated or not with 50units/ml IFN**γ** type II *Toxoplasma* for 2.5h before fixation and staining with α-Galectin 8 for fluorescence microscopy. Galectin 8 positive vacuoles were counted in >100 vacuoles. The mean of 3 experiments is shown. Significance was determined by 2way ANOVA, ns, not significant. (C) Representative confocal images of galectin 8 staining type II *Toxoplasma* vacuoles 2.5h p.i.. Scale bar 10μm.
(TIF)

S9 Fig Atg16L1 is knocked down in HUVEC by siRNA. (A) Immunoblot showing lysates of HUVEC cells treated with siRNA control and Atg16L1 and probed with antibody to Atg16L1. Loading control is shown with antibody to β-actin. (B) Immunoblot showing lysates from HUVEC treated or not with 100nM rapamycin for 24h and with and without 400nM bafilomycin A1 for 2h post rapamycin treatment. Antibodies to LC3B and p62 were used to probe the blots. Control for loading controls was assessed by β-actin antibody staining.
(TIF)

S10 Fig Rab7 is recruited to the type II *Toxoplasma* vacuole in IFN0γ-stimulated HUVEC. Representative confocal images of Rab7 staining type II *Toxoplasma* vacuoles 2.5h p.i.. Scale bar 10μm.
(TIF)

S11 Fig LAMP1 and K63 linked ubiquitin are present on the type II vacuole in IFNγ-stimulated HUVEC. Movie of consecutive 0.4μm confocal z stacks of the type II PV, in IFNγ-stimulated HUVEC, co-stained with LAMP1 (red) and K63 ubiquitin (white) antibodies, is shown, with *Toxoplasma* (green), Hoechst (blue).
(MOV)

S12 Fig LAMP1 and K63 linked ubiquitin are present on the type II vacuole in IFNγ-stimulated HUVEC. Movie of consecutive 0.4μm confocal z stacks of the type II PV, in IFNγ-stimulated HUVEC, co-stained with LAMP1 (red) and K63 ubiquitin (white) antibodies, is shown, with *Toxoplasma* (green), Hoechst (blue).
(MOV)

S13 Fig Electron micrographs of type II *Toxoplasma* in IFNγ-stimulated HUVEC showing parasite degradation inside the PV. Representive images of digested parasites inside their own PV are shown. Many more degraded parasites were observed in IFN**γ**-stimulated cells containing type II parasites compared with type I parasites. Arrows indicate vacuoles containing degraded parasites.
(TIF)

S14 Fig Tryptophan supplementation does not increase the replicative capacity of *Toxoplasma* in HUVEC. HUVEC that had been IFNγ stimulated for 18h were infected, with or without the addition of 1mM L-tryptophan, allowing the infection to continue for 24h. The cells were then fixed and the number of vacuoles containing replicated *Toxoplasma* was counted using immunofluorescence microscopy. Mean from 3 experiments shown. Significance was determined using 2way ANOVA, ns, not significant.
(TIF)

S1 Table Comparison of recruitment of proteins to the PV of HUVEC and HeLa cells. Percentage recruitment of protein markers to the type II *Toxoplasma* PV of IFNγ-stimulated HUVEC and HeLa are compared between this paper and [26]. Times post infection are indicated.
(TIF)

S1 Appendix Extended methods for immunofluorescence staining, superresolution SIM microscopy and electron microscopy (DOCX)
