# Supplementary figures and images for: K63-Linked Ubiquitination Targets Toxoplasma gondii for Endo-lysosomal Destruction in IFNγ-Stimulated Human Cells
Source: PLoS Pathog. 2016 Nov 22;12(11):e1006027. doi: 10.1371/journal.ppat.1006027 (PMC5119857; doi:10.1371/journal.ppat.1006027)

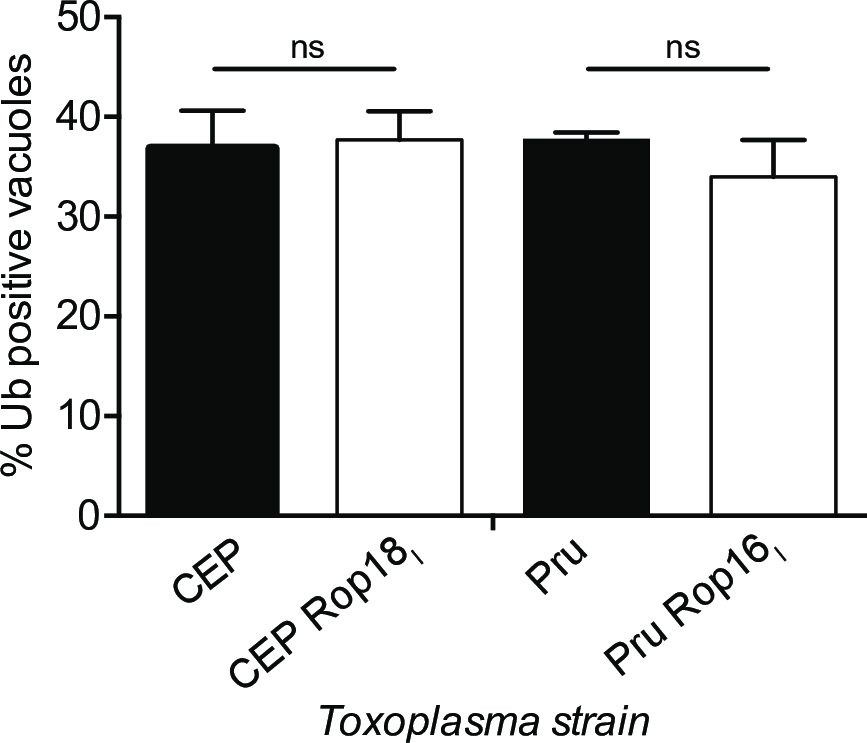

Supplement: S3 Fig — HUVEC stimulated or not with 50units/ml IFNγ type II (Pru) and type III (CEP) virulence mutants CEP-ROP18I and Pru-ROP16I and their controls for 2.5h before fixation for fluorescence microscopy. Ubiquitin positive vacuoles were counted in >100 vacuoles. The mean of 3 experiments is shown. Significance was determined by 2way ANOVA, ns, not significant. (TIF) [file ppat.1006027.s003.tif]

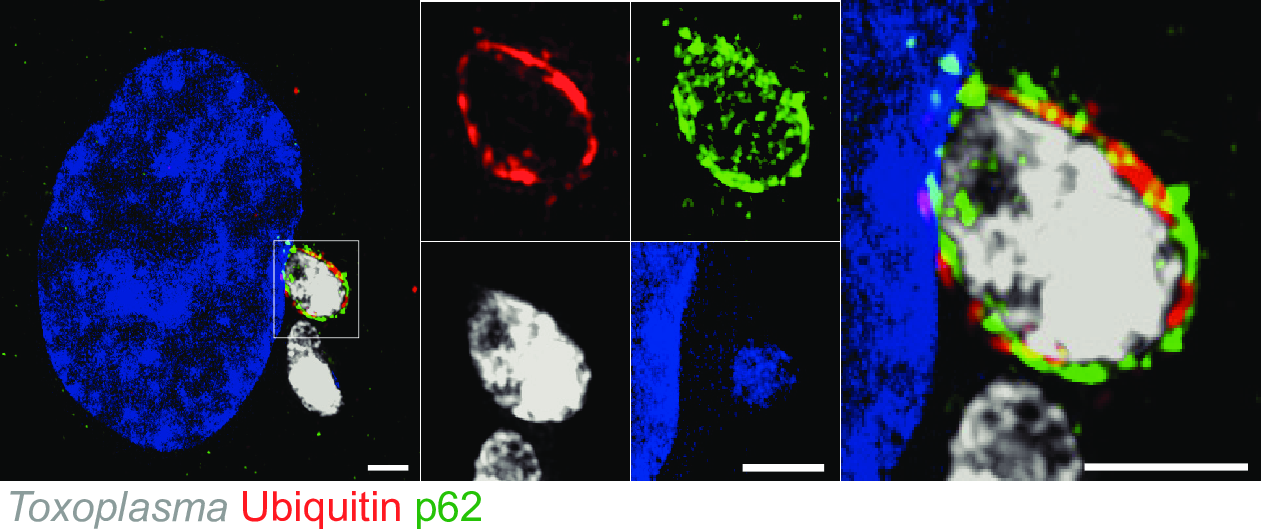

Supplement: S6 Fig — Superresolution Structured Illumination Microscopy image of ubiquitin and p62 co-staining of type II PV. Total ubiquitin (red), p62 (green), Toxoplasma (white) and Hoechst (blue) are shown. Scale bar 2μm (TIF) [file ppat.1006027.s006.tif]

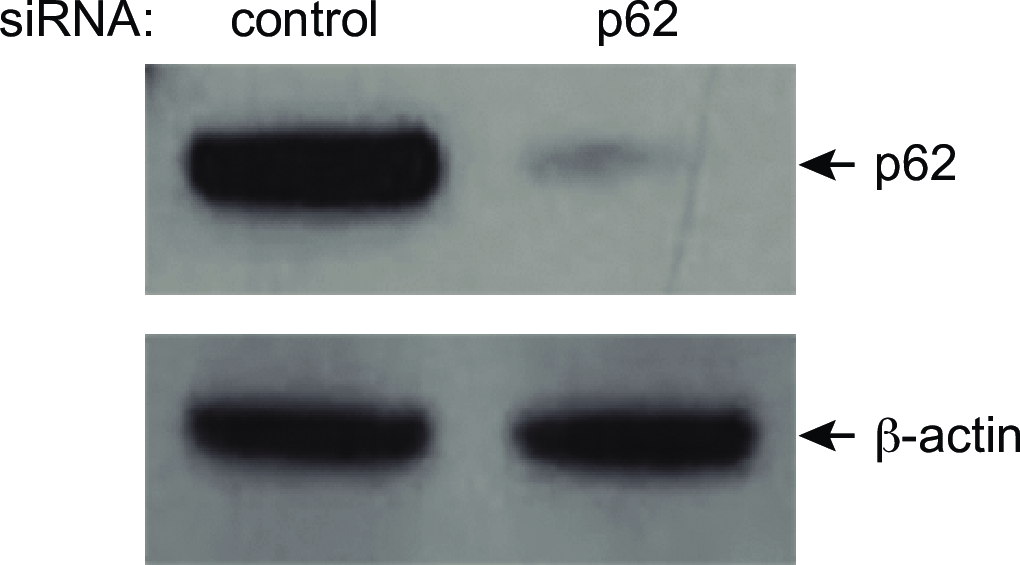

Supplement: S7 Fig — Immunoblot showing lysates of HUVEC cells treated with siRNA control and p62 and probed with antibody to p62. Loading control is shown with antibody to β-actin. (TIF) [file ppat.1006027.s007.tif]

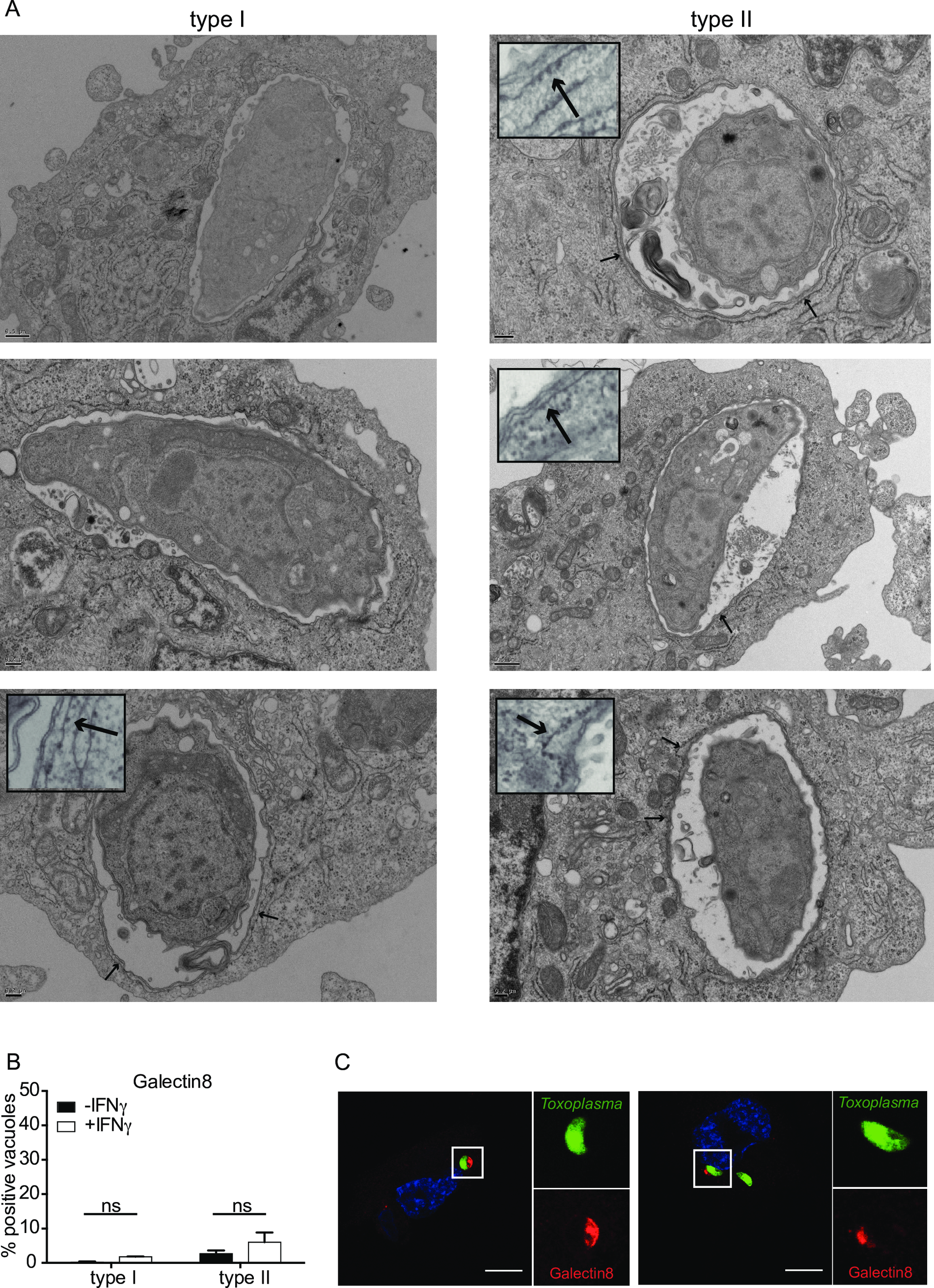

Supplement: S8 Fig — (A) Additional electron micrographs all demonstrate that the PVs containing type I or type II Toxoplasma do not break in IFNγ-stimulated HUVEC. Arrows indicate rough endoplasmic reticulum closely apposed to the vacuoles of both type I and type II parasites, enlarged view in boxes. Scale bar = 0.2μm (or 0.5μm centre left and top right). (B) HUVEC stimulated or not with 50units/ml IFNγ type II Toxoplasma for 2.5h before fixation and staining with α-Galectin 8 for fluorescence microscopy. Galectin 8 positive vacuoles were counted in >100 vacuoles. The mean of 3 experiments is shown. Significance was determined by 2way ANOVA, ns, not significant. (C) Representative confocal images of galectin 8 staining type II Toxoplasma vacuoles 2.5h p.i.. Scale bar 10μm. (TIF) [file ppat.1006027.s008.tif]

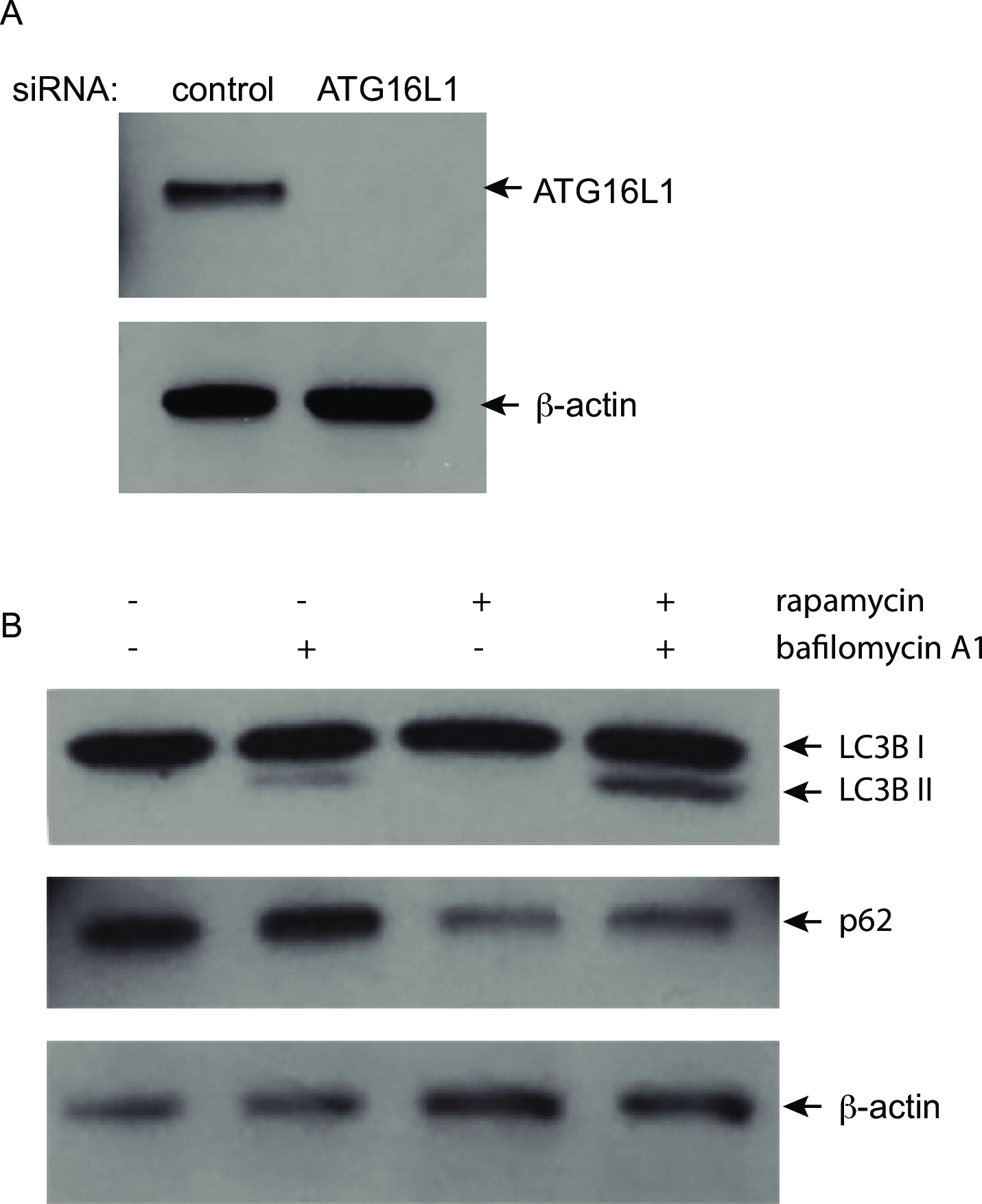

Supplement: S9 Fig — (A) Immunoblot showing lysates of HUVEC cells treated with siRNA control and Atg16L1 and probed with antibody to Atg16L1. Loading control is shown with antibody to β-actin. (B) Immunoblot showing lysates from HUVEC treated or not with 100nM rapamycin for 24h and with and without 400nM bafilomycin A1 for 2h post rapamycin treatment. Antibodies to LC3B and p62 were used to probe the blots. Control for loading controls was assessed by β-actin antibody staining. (TIF) [file ppat.1006027.s009.tif]

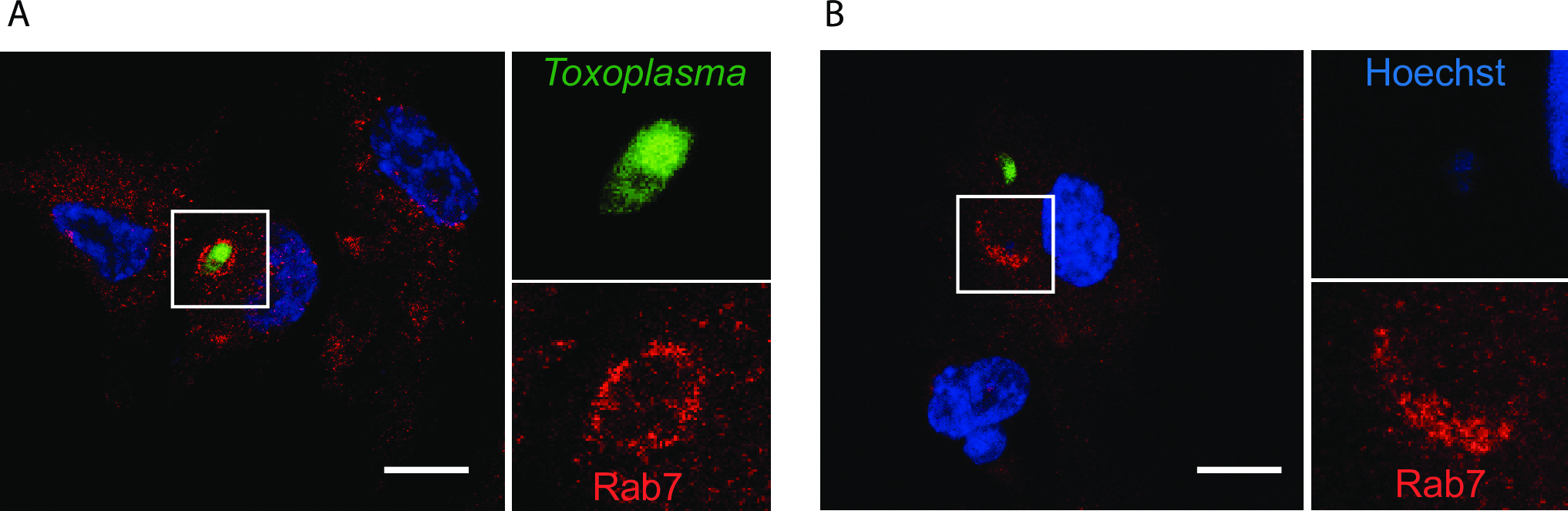

Supplement: S10 Fig — Representative confocal images of Rab7 staining type II Toxoplasma vacuoles 2.5h p.i.. Scale bar 10μm. (TIF) [file ppat.1006027.s010.tif]

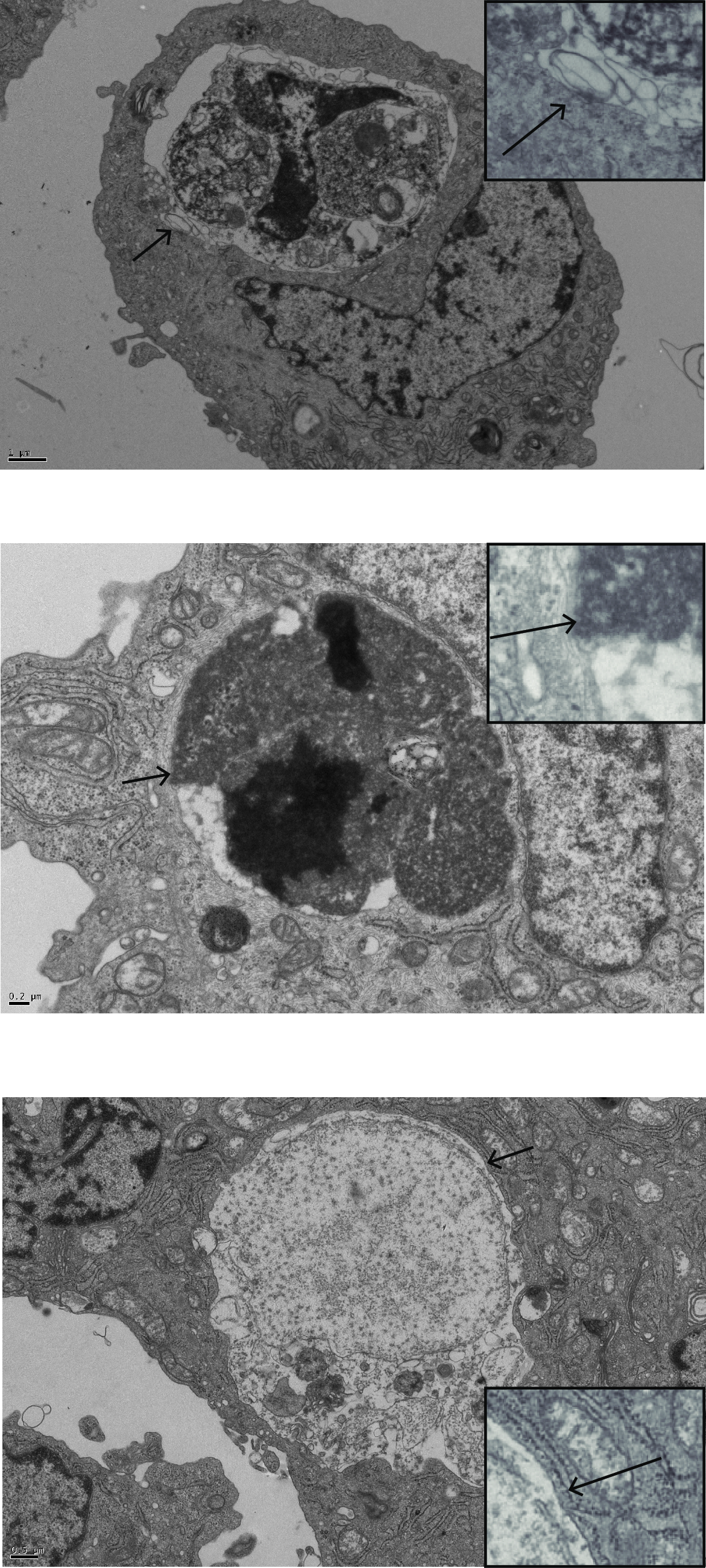

Supplement: S13 Fig — Representive images of digested parasites inside their own PV are shown. Many more degraded parasites were observed in IFNγ-stimulated cells containing type II parasites compared with type I parasites. Arrows indicate vacuoles containing degraded parasites. (TIF) [file ppat.1006027.s013.tif]

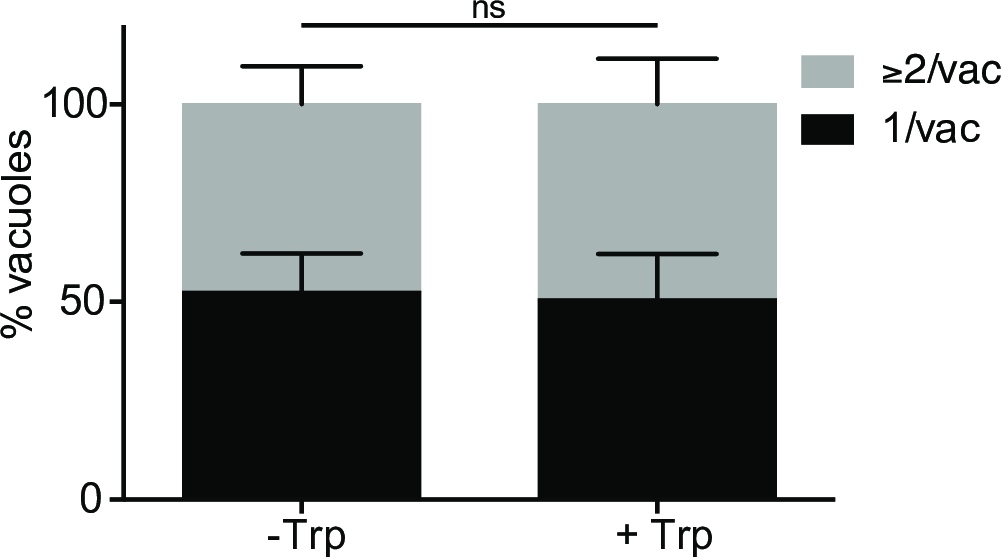

Supplement: S14 Fig — HUVEC that had been IFNγ stimulated for 18h were infected, with or without the addition of 1mM L-tryptophan, allowing the infection to continue for 24h. The cells were then fixed and the number of vacuoles containing replicated Toxoplasma was counted using immunofluorescence microscopy. Mean from 3 experiments shown. Significance was determined using 2way ANOVA, ns, not significant. (TIF) [file ppat.1006027.s014.tif]

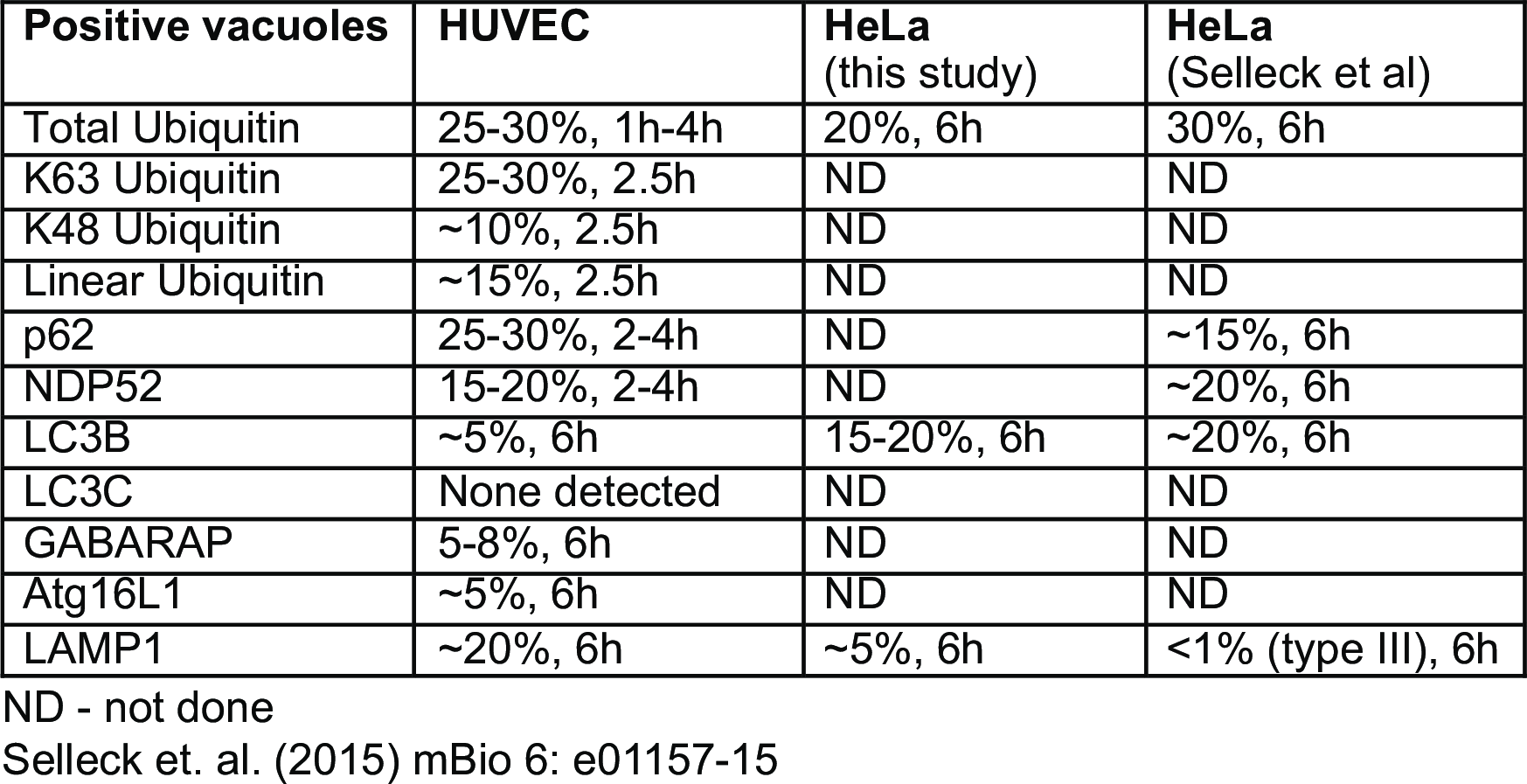

Supplement: S1 Table — Percentage recruitment of protein markers to the type II Toxoplasma PV of IFNγ-stimulated HUVEC and HeLa are compared between this paper and [26]. Times post infection are indicated. (TIF) [file ppat.1006027.s015.tif]
