# Supplementary material for: K63-Linked Ubiquitination Targets Toxoplasma gondii for Endo-lysosomal Destruction in IFNγ-Stimulated Human Cells
Source: PLoS Pathog. 2016 Nov 22;12(11):e1006027. doi: 10.1371/journal.ppat.1006027 (PMC5119857; doi:10.1371/journal.ppat.1006027)
Supplement: S1 Appendix — (DOCX) [file ppat.1006027.s016.docx]

**S1 Appendix**

**Materials and Methods**

Fixed immunofluorescence microscopy

All following steps were carried out at RT. The cells mounted onto coverslips were washed 3x with PBS to remove uninvaded *Toxoplasma* and fixed with 3% paraformaldehyde (Sigma) in PBS for 20 mins. The fix was aspirated, washed 1x with Perm-Quench solution (50mM NH_4_Cl, 0.2% w/v saponin, (Sigma 47036) in PBS) before incubation with fresh Perm-Quench for 10-15 min. The Perm-Quench solution was replaced with PGAS (0.2%w/v fish gelatin, Sigma G-7765, 0.02% w/v saponin, 0.02% w/v NaN_3_ in PBS) and incubated for at least 5mins. Fixed cells could be stored in PGAS at 4^o^C for several days. Antibody incubations were carried out in a humid box, inverting coverslips onto 50μl drops of primary antibody, diluted in PGAS, and incubating for 1h at RT. Coverslips were washed in 3 x 1ml volumes of PGAS before incubating for a further 1h with second antibody, diluted in PGAS, at RT in the dark. Washes of 3 x 1ml PGAS and 2 x 1ml PBS followed by 1ml PBS containing 1μg/ml Hoechst 33342 (Life Technologies) and finally 2 washes in dH_2_O prior to mounting on glass slides with Mowiol 4-88 (Polysciences Inc.). Mounting medium was allowed to harden overnight.

For LC3 and GABARAP staining the following protocol was used and carried out at room temperature. Cells mounted on coverslips were washed 3x with PBS to remove uninvaded *Toxoplasma* and fixed for 20 mins in 3% paraformaldehyde (Sigma) in PBS. Coverslips were washed 1x in PBS and room temperature methanol added for 5mins. After washing 2x in PBS 5% BSA (Fraction V) in PBS was added for 1h as a blocking agent. Antibody incubations were carried out in a humid box, inverting coverslips onto 50μl drops of primary antibody, diluted in 1%BSA in PBS for 1h. Washes of 3x 1ml PBS were made before incubating for a further 1h with second antibody diluted in 1 %BSA in PBS at RT in the dark. Coverslips were then washed 2 x 1ml PBS followed by 1ml PBS containing 1μg/ml Hoechst 33342 (Life Technologies) and finally 2 washes in dH_2_O prior to mounting on glass slides with Mowiol 4-88 (Polysciences Inc.). Mounting medium was allowed to harden overnight.

Slides were viewed on a Zeiss Axioplan II Epifluorescence microscope using x100 objective, imaged with an AxioCam HRC camera and analysed with Axiovision 4.8 software or on an SP5-invert Confocal microscope using x63 or x100 objective and analysed using LAS-AF software.

Superresolution Structured Illumination Microscopy (SR-SIM)

Images were acquired using 5 phase shifts and 3 grid rotations, with 34µm grating period for the 647nm and 561nm lasers, 28µm grating period for the 488nm laser and 23µm grating period for the 405nm laser and filter set 3 (1850-553, Zeiss).

Transmission electron microscopy

After centrifugation at 900g for 5 min, cells were immersion fixed in 2% glutaraldehyde (Agar Scientific)/2% paraformaldehyde in 0.1M sodium cacodylate buffer, pH7.2, embedded in 2% agarose and centrifuged for 3 min at 13000rpm before being returned to 2% glutaraldehyde/2% paraformaldehyde in 0.1M sodium cacodylate buffer, pH7.2 overnight. Samples were washed in sodium cacodylate buffer 0.1M pH7.2 (SCB) for 10 min, post-fixed in 1% osmium tetroxide/SCB for 1.5h, washed in SCB for 10 min and stained on bloc in 1% aqueous uranyl acetate for 1.5h. Samples were dehydrated in 50% ethanol, 75% ethanol and 90% ethanol for 10 min each, 3x 10 min in 100% ethanol and 2x 30 min in propylene oxide. Samples were embedded in epon resin (Agar Scientific, Stansted, UK) for 5 changes over 8h and polymerised at 70°C overnight. 50nm sections were mounted on pioloform-coated slot grids and stained with uranyl acetate for 30 min and with Reynold’s lead citrate for 7 min.
